# An Umbrella Review and Updated Meta-Analysis of Imaging Modalities in Occult Scaphoid and Hip and Femoral Fractures

**DOI:** 10.3390/jcm13133769

**Published:** 2024-06-27

**Authors:** Patricia Jorisal, Callistus Bruce Henfry Sulay, Gilbert Sterling Octavius

**Affiliations:** 1 Abdominal Radiology, Department of Radiology, Faculty of Universitas Pelita Harapan, Tangerang 12930, Indonesia; 2 Department of Radiology, Siloam Hospital Kebon Jeruk, Jakarta 11530, Indonesia; 3 Radiology Resident, Faculty of Universitas Pelita Harapan, Tangerang 12930, Indonesia

**Keywords:** occult fracture, magnetic resonance imaging, computed tomography, scaphoid, hip

## Abstract

**Background:** Occult fractures may cause multiple morbidities. If occult fractures were detected earlier, complications may be preventable. This umbrella review and updated meta-analysis will aim to evaluate the use of imaging modalities in detecting occult scaphoid and hip fractures. **Methods:** The protocol for this study is available in the International Prospective Register of Systematic Reviews (PROSPERO) database (CRD42024525388). The literature search started and ended on 17 March 2024. We searched seven academic databases: MEDLINE, Cochrane Library, Pubmed, Science Direct, Google Scholar, WHO International Clinical Trials Registry Platform, and The Joanna Briggs Institute (JBI) database. The meta-analysis was conducted with the STATA program using the “midas” command. **Results:** There are four systematic reviews evaluating occult hip and femoral fractures with 6174 patients and two reviews evaluating occult scaphoid fractures with 1355 patients. The prevalence of occult scaphoid fracture and occult hip and femoral fractures is 23.87% (95% CI 18.25–29.49) and 44.8% (95% CI 39.38–51.4), respectively. Magnetic resonance imaging (MRI) had the best posterior probability of positive likelihood ratio (LR+) with 95% and 96% and negative likelihood ratio (LR-) with 0.15% and 1% for both occult scaphoid and hip fractures, respectively, assuming a 25% baseline. MRI could both confirm and exclude occult hip fractures while it can only confirm occult scaphoid fractures. Bone scans are inappropriate for either type of occult fractures The level of evidence for occult scaphoid fracture is weak while it is suggestive for occult hip fractures. **Conclusion:** The findings strengthen the use of MRI after an initially negative radiograph fracture for occult hip and femoral fractures, with a CT scan as a viable second option.

## 1. Introduction

The definition of occult fractures, or negative radiograph fractures, is a fracture that is not initially seen in all available conventional radiography projections and a follow-up imaging (using conventional radiography or other modalities) reveals the cortical break in the bones [1]. Delayed diagnosis and treatment increase the likelihood of complications such as nonunion, avascular necrosis, and osteoarthritis in occult scaphoid fractures. Patients will also require further imaging while being splinted. This overtreatment results in a loss of productivity for the patients and a potential lawsuit for the clinical provider [2,3]. Although there is no increased mortality in occult hip fractures as compared to patients with apparent radiograph hip fractures [4], delaying the diagnosis of a proximal femur hip fracture can result in poorer patient outcomes. Earlier diagnosis of hip and femoral fractures will obviate the need for more extensive surgery [5].

The use of additional imaging for occult fractures is not new. However, the debate on what is the best next course of action after an initial negative radiograph persists. The American College of Radiology (ACR) recommends magnetic resonance imaging (MRI) as the first line for detection of occult fractures. However, some authors argue against the routine use of MRI in the detection of occult fractures as it leads to overdiagnosis and overtreatment [6]. Another point of view is that the patient’s preferences need to be incorporated into the decision-making, which hinges the diagnosis on multifactorial components and not just on the sensitivity and specificity of said imaging modalities [7].

There are also other modalities with strengths and limitations in diagnosing occult fractures, which may complement each other. For example, computed tomography (CT) may also replace MRI due to its more widespread availability in smaller centres compared to MRI despite its radiation penalty. There is also ultrasound, which is gaining increasing traction to be used as a radiation-free and widely available bedside tool to diagnose occult fracture, although the literature is very scarce. Lastly, nuclear studies or bone scans are very sensitive but the relative radiation level is sometimes unacceptable [8,9].

Therefore, this umbrella review and updated meta-analysis will aim to evaluate the use of imaging modalities in detecting occult scaphoid and hip fractures. To the best of our knowledge, this is the first attempt at an umbrella review and updated meta-analysis in this field, which will comprehensively summarize all the literature available on the pertaining topic.

## 2. Materials and Methods

### 2.1. Search Strategy and Study Selection

The literature search started and ended on 17 March 2024. We searched five academic databases: MEDLINE, Cochrane Library, Pubmed, Science Direct, and Google Scholar. The authors also searched the WHO International Clinical Trials Registry Platform and The Joanna Briggs Institute (JBI) database on the same day for ongoing clinical trials. The keywords used were related to the diagnostic tool (“X-ray, “cone beam computed tomography”, “magnetic resonance imaging”, “computed tomography”, “bone scan”, and “ultrasound”) and the condition under study (“occult fracture”, “suspected fracture”, “non-displaced fracture”, and “negative radiograph fracture”), as well as the word or filter “systematic review” and “meta-analysis”. Supplementary Table S1 lists the Medical Subject Heading (MeSH) terms for each database. All records were entered into the Rayyan program, which manually screened them and automatically identified duplicates [10]. The initial search was conducted by authors GSO and CBHS, who imported all findings into Rayyan software. A separate evaluation of the initial searches was performed by author P.J. Each paper underwent individual assessment by GSO and CBHS, with conflicts resolved through group discussion and professional judgment from P.J. In instances where research from the same dataset had overlapping time points, we prioritized data that offered the most comprehensive information.

After the umbrella review was conducted, we found two anatomical sites of occult fractures included in our review, which were the hip or femur and scaphoid. A separate search strategy was conducted on the same day (25 March 2024) for these two anatomical sites using the same strategy as above and the MeSH terms were listed in Supplementary Tables S2 and S3. There are no other anatomical sites that could be included in this review which met the criteria. The search of the study was limited to one year before the latest systematic review until the present. For example, if the latest systematic review on scaphoid searched for 2020, we limited the search term from 2019 until 25 March 2024. 

### 2.2. Eligibility Criteria

The authors followed the Preferred Reporting Items for Systematic reviews and Meta-Analyses (PRISMA) 2020 as well as the PRISMA Diagnostic Test Accuracy (PRISMA-DTA) guidelines [11,12]. The protocol for this study is available in the International Prospective Register of Systematic Reviews (PROSPERO) database (CRD42024525388).

This is an umbrella review and although the methodology had not been properly standardized [13,14], this review opted for an updated meta-analysis [15]. Therefore, the authors conducted a systematic search according to PRISMA 2020 guidelines for each topic found under the umbrella review. The studied population was all patients with occult fractures (defined as no cortical breaks or fracture lines seen on a plain radiograph series with a high clinical suspicion for fracture due to intense pain, swelling, inability to move the affected body parts, or other clinical suspicions) who underwent further imaging with reference standards. In this review, reference standards were considered adequate if they fulfilled one of the following criteria: (1) A follow-up plain radiograph series (with a minimum of two views) conducted at a minimum of six weeks apart after the initial injury; (2) clinical findings with an index test or repeated radiographs to formulate a reference standard; and (3) the use of advanced imaging that serves as a reference standard, which differs from the modality used as the index test (i.e., the use of a single computed tomography [CT] or MRI without any referenced index would not suffice). In a retrospective study, this review also considered studies that confirmed fractures with a clinical follow-up only, although this is not optimal. 

### 2.3. Inclusion and Exclusion Criteria

The inclusion criteria were articles of any prospective or retrospective cohort studies that studied occult fractures in all ages with a reference index as delineated above. The search was also conducted for grey literature, including theses, dissertations, and conference abstracts. The exclusion criteria included case series, case reports, or animal research. Studies that did not perform the reference test according to our criteria were also excluded. To ensure that the literature was saturated, review study citations were looked up. We also manually searched and cited the literature to make sure that all pertinent studies were covered. There was no restriction on the language.

### 2.4. Data Extraction and Quality Assessment

Two authors (G.S.O. and C.B.H.S.) independently extracted the data, while all three authors verified its accuracy. We gathered pertinent data, including study identity (author and publication year), study characteristics (location, study design, and age of the participants), modalities used for the imaging, and the operational definition of fractures. The number of true positives (TP), false positives (FP), true negatives (TN), and false negatives (FN) were also extracted from each study. 

As for the umbrella review, the A MeaSurement Tool to Assess Systematic Reviews 2 (AMSTAR 2) was used to evaluate each systematic review. A high-quality systematic review has no or one non-critical weakness, a moderate-quality systematic review means there is more than one non-critical weakness. One critical flaw with or without non-critical weakness placed the systematic review in low quality, while a critically low systematic review meant that there was more than one critical flaw with or without non-critical weakness [16]. 

To evaluate the risk of bias in the updated meta-analysis, we employed the Revised Tool for the Quality Assessment of Diagnostic Accuracy Studies (QUADAS-2). In QUADAS-2, there are no official cut-off scores, and bias risk is displayed graphically [17].

The scale was independently assessed by two reviewers, G.S.O. and C.B.H.S., with any discrepancies resolved internally and through the expertise of P.J. until consensus was achieved. In cases of missing or incomplete data, we reached out to the associated authors via email.

### 2.5. Stratification of Evidence

After the updated meta-analysis, a stratification of evidence was conducted according to this classification: Class I: Convincing—When there are over 1000 cases, the *p*-value is less than 10^−6^, I2 is less than 50%, 95% prediction interval excludes the null hypothesis, there are no small-study effects, and no excess significance bias; Class II: Highly suggestive—When there are over 1000 cases, *p*-value is less than 10^−6^, the largest study shows a statistically significant effect, but it does not meet all Class I criteria; Class III: Suggestive—When there are over 1000 cases, *p*-value is less than 10^−3^, and it does not meet Class I or II criteria; Class IV: Weak—When *p*-value is less than 0.05, but it does not meet Class I to III criteria; and Non-significant—When *p*-value is greater than 0.05 [14].

### 2.6. Data Synthesis

This review evaluated matrices of evidence and the calculation of the overall corrected covered area (CCA) according to Bracchiglione et al. [18] Each study in a given systematic review would be read one by one to ensure that they fulfil the inclusion criteria. This review calculated the point prevalence of occult fractures by dividing the number of occult fractures by the total number of patients being worked up for occult fractures. DerSimonian and Laird’s random-effect model was chosen, and we calculated the 95% confidence interval (CI) using the Clopper–Pearson method [19]. We used prediction intervals to assess heterogeneity if more than 10 studies were included [20], and between-study heterogeneity was explored with a Galbraith plot [21]. Small-study effects were assessed with funnel plot analysis if there were more than ten studies included [22], Begg and Mazumdar’s test for rank correlation [23], and Egger’s test for a regression intercept [24]. Trim-and-fill analysis was done if there was an asymmetry in the funnel plot [25]. We also conducted a sensitivity analysis to assess one study’s impact on the overall prevalence [26].

The sensitivity, specificity, LR+, LR−, PPV, and NPV of each study were calculated if no information was given in the article [27]. Model diagnostics were verified using a graphical representation of residual-based goodness of fit, bivariate normality, influence, and outlier detection analysis before moving forward with the meta-analysis. Sensitivity analyses would be used to confirm any outlier research. The level of interconnectedness would be represented by the bivariate box plot. We used the bivariate model of sensitivity and specificity to calculate the individual and pooled sensitivity and specificity [28]. Using the hierarchical summary receiver operating characteristic (HSROC), a summary receiver operating characteristic (SROC) was utilized to show and highlight the trade-off between sensitivity and specificity [29]. The area under the curve (AUC) was measured, and a value of 0.9–1 indicated excellent diagnostic accuracy, 0.8–0.9 indicated very good diagnostic accuracy, 0.7–0.8 indicated good diagnostic accuracy, 0.6–0.7 indicated sufficient diagnostic accuracy, and 0.5–0.6 meant poor diagnostic accuracy [30]. Heterogeneity was measured using the I2 index; a value of 0% indicated that there was no detectable heterogeneity, while a value of more than 50% was considered to be high heterogeneity [31]. To assess publication bias, a linear regression test of funnel plot asymmetry was performed; a slope coefficient of less than 0.1 signified a significant asymmetry. We used the Fagan plot, which is based on the likelihood ratio scattergram and the Bayes theorem, to determine the post-test probability. An arbitrary cut-off point was set at <−0.1 for LR− and >+10 for LR+, indicating significant changes in the probability ratio [32]. Furthermore, the probability modifying plots and predictive values were shown; curves that incline toward the (0,1) location were produced by test results with more informative positive results, and curves that incline toward the (1,0) line were produced by test results with more informative negative results [33]. The meta-analysis was carried out using the STATA program (Version 17.0, StataCorp, College Station, Texas, USA) using the “midas” commands. These commands could only synthesize data if there were at least four studies included [34].

## 3. Results

**Figure 1:**
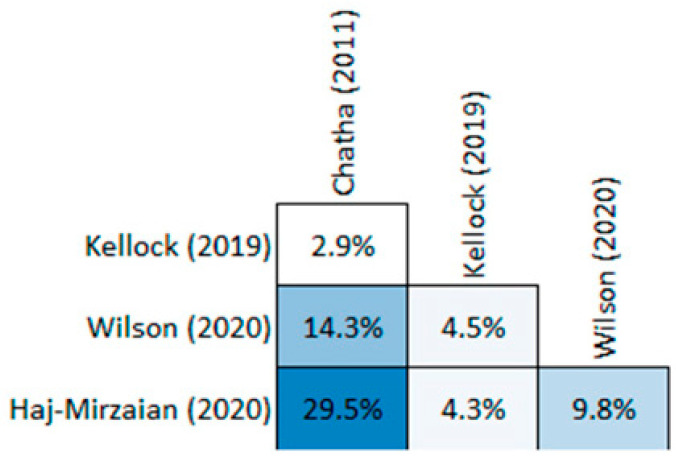
Graphical Representation of Overlap for OVErviews (GROOVE) of occult hip and femur fractures [35,36,37,38].

A total of 12,488 articles were initially identified, out of which 40 duplicates were promptly eliminated, leaving 12,448 unique articles for screening. Following the evaluation of titles and abstracts, 12,136 articles were excluded, and ultimately, six studies were included in the umbrella review (Supplementary Figure S1). Four reviews [35,36,37,38] evaluated occult hip and femur fractures with a combined total of 6174 patients and two reviews [39,40] evaluated occult scaphoid fractures with a combined total of 1355 patients. Amongst reviews evaluating hip and femur fractures, three of them achieve a high quality under AMSTAR-2 scoring, while one scores a critically low rating. Amongst scaphoid reviews, one scores a high quality and one scores a critically low rating (Table 1). There is a high overlap (12.94%) between the primary studies of occult hip and femur fractures, with 16 overlapping studies in two systematic reviews and three studies in three systematic reviews. The review conducted by Haj-Mirzaian (2020) [38] and Chatha (2011) [35] produced the largest overlap with 29.5% (Figure 1). Notable exclusions are presented in Supplementary Table S4.

**Figure 2:**
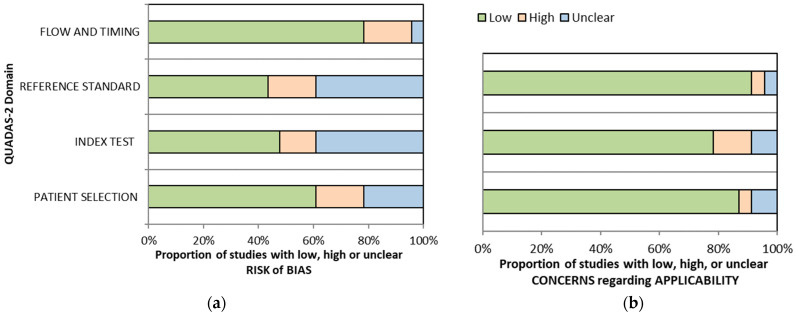
QUADAS-2 graphical representation of the risk-of-bias (**a**) and concerns regarding applicability (**b**) of occult scaphoid fractures.

An additional search for scaphoid occult fractures yielded four new articles [41,42,43,44] (Supplementary Figure S2). There were 23 studies with 1208 patients with suspected occult scaphoid fractures and 236 patients with eventual scaphoid fractures (Supplementary Table S7). Notable exclusions are presented in Supplementary Table S5. One study [45] was removed from a meta-analysis included in the umbrella review [40] as it did not fit the inclusion criteria. All studies were done prospectively with only thirteen studies explicitly mentioning that the studies employed consecutive sampling. Six studies [46,47,48,49,50,51] used ultrasound as the index test enrolling 270 patients, five studies [41,52,53,54,55] used a CT scan enrolling 297 patients, eight studies [42,43,44,54,55,56,57,58] used MRI enrolling 345 patients, seven studies [44,52,56,59,60,61,62] used bone scans enrolling 586 patients, and two studies [63,64] used cone-beam computed tomography (CBCT) enrolling 144 patients. Most patients are male, with females only ranging from 0% [53] to 64% [43]. The reference test was widely heterogeneous, ranging from retrospective clinical examination, a follow-up X-ray, bone scan, CT scan, MRI, or a combination of clinical and radiographic examinations. The machines and protocols used also varied widely (Supplementary Table S6). As a whole, the studies included for occult scaphoid fracture had relatively good qualities. However, only four studies [47,48,50,57] were free of bias, while the rest were at risk of some bias (Figure 2 and Table A1). 

The prevalence of occult scaphoid fracture is 23.87% (95% CI 18.25–29.49) (Supplementary Figure S3). The prediction interval, representing an absolute measure of heterogeneity, exhibits a prevalence rate of 17.8% to 30.2%. The Galbraith plot indicates some heterogeneity (Supplementary Figure S4). Begg and Mazumdar’s test for rank correlation gives a *p*-value of <0.001, indicating possible evidence of publication bias. Egger’s test for a regression intercept gives a *p*-value of 0.0005, indicating possible evidence of publication bias. The funnel plot indicates a small-studies effect as the results show asymmetry, not corrected by trim-and-fill (Supplementary Figure S5).

Bone scan had the highest sensitivity (98%; 95% confidence interval [CI] 90–100%) while CT scan (98%; 95% 86–100) had the highest specificity. Although MRI also has a 98% specificity, the 95% CI is much wider than CT scan. In this meta-analysis, MRI had the best posterior probability of LR+ and LR− amongst other imaging modalities, with 95% and 0.15%, respectively, assuming a baseline 25% prior probability. Heterogeneity exists amongst all imaging modalities, except for bone scan (I2 = 1 and *p*-value of 0.182). Publication bias does not exist for studies including ultrasound and bone scan. According to the likelihood ratio scattergram, ultrasound can be used as an exclusion and confirmation imaging modality, CT and MRI can only be used for confirmation of occult fracture, while bone scan can neither exclude nor confirm occult fracture. All of the imaging modalities have an excellent AUC, with bone scans having the best AUC value of 0.99 (95% CI 0.97–0.99). Although ultrasound has an AUC of 0.99, the 95% CI is very wide (0.2–1) (Supplementary Figure S6A–D). All of the imaging modalities only have weak evidence (Table 2).

There are only two eligible studies that studied CBCT as their index test, hence the results will be qualitatively studied [63,64]. Both studies used MRI as their reference standard, with a total of 144 studies across both studies. Sensitivity is lower in Edlund’s [64] study with 69% (95% CI 41–88%) as compared to Borel’s [63] study with 100% (95% CI 75–100%). However, it should be noted that Edlund employed the MRI scan two weeks after the initial fracture, while Borel conducted the MRI scan one week after the initial fracture. Borel also found that CBCT had a specificity of 95% (95% CI: 75–100%), PPV of 96% (95% CI: 78–100%), and NPV of 100% (95% CI: 83–100%) [63]. Both studies conclude that CBCT is superior to radiography, but Edlund noted that CBCT cannot be used to exclude scaphoid fractures.

The model diagnostics are shown in Supplementary Figure S7A–D, where no studies appear to be outliers, as evidenced by the outlier detection, with a reasonable goodness-of-fit for MRI and bone scan studies. The goodness-of-fit is not linear in USG and CT studies. The bivariate normality assumption is not fulfilled, and some studies [41,44,46,47,49,52,53] seem to be more influential than others with a Cook’s distance of >0.5. The bivariate box plot showed a skewness of the test performance measures toward a higher sensitivity with lower specificity for ultrasound, and vice versa for CT, MRI, and bone scan, providing indirect evidence of some threshold variability (Supplementary Figure S8A–D). Supplementary Figure S9A–D presents the paired forest plot description of empirical Bayes predicted versus observed study-specific sensitivity and specificity, indicating threshold variability as sensitivity increases and specificity decreases and vice versa. 

**Figure 3:**
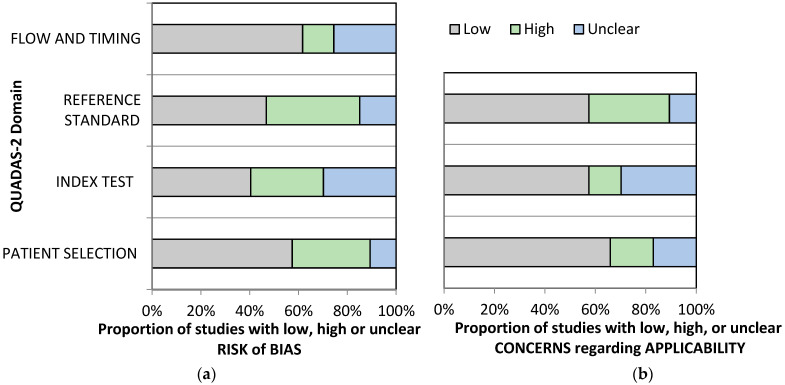
QUADAS-2 graphical representation of the risk-of-bias (**a**) and concerns regarding applicability (**b**) of occult hip and femoral fractures.

The updated meta-analysis for occult hip and femur fracture yields an additional two articles [65,66], with three hand-searched articles [67,68,69]. Hence, there are five more new articles included in this updated meta-analysis (Supplementary Figure S10). Two [70,71] out of three [72] articles included in previous systematic reviews [36,38] had to be excluded as we believe the cohort is too similar. There were 45 studies with 3594 patients with suspected occult scaphoid fractures and 1523 patients with eventual scaphoid fractures (Supplementary Table S7). Notable exclusions are presented in Supplementary Table S8. Eleven studies were done prospectively with 29 studies being done retrospectively. The rest of the studies did not provide sufficient or direct information regarding the types of studies. Only fifteen studies explicitly mentioned that the studies were done consecutively, one being done non-consecutively, and the rest provided no information. Two studies [67,73] used ultrasound, three studies [74,75,76] employed bone scan as their index test, fifteen studies [65,66,68,69,77,78,79,80,81,82,83,84,85,86,87] used CT scans and the rest used MRI [72,74,75,77,78,88,89,90,91,92,93,94,95,96,97,98,99,100,101,102,103,104,105,106,107,108,109,110,111]. Most patients were female, ranging from 25% [95] to 95% [90] The reference test was heterogeneous, but most studies employed clinical and surgical follow-up. Only eight studies used MRI [65,67,68,73,76,82,87,88] as the reference test. The machines and protocols used also varied widely. As a whole, the studies included for occult hip and femoral fracture suffer from a moderate to high risk of bias and concerns of applicability. Only eight studies [72,73,82,87,106,108,110,111] were free of bias, while the rest were at risk of some bias (Figure 3 and Table A2).

The prevalence of occult hip and femoral fractures is 44.8% (95% CI 39.38–51.4) (Supplementary Figure S11). The prediction interval, representing an absolute measure of heterogeneity, exhibits a prevalence rate of 38.2% to 51.4%. The Galbraith plot indicates some heterogeneity (Supplementary Figure S12). Begg and Mazumdar’s test for rank correlation gives a *p*-value of 0.09, indicating little evidence of publication bias. Egger’s test for a regression intercept gives a *p*-value of 0.0235, indicating possible evidence of publication bias. The funnel plot indicates a small-studies effect as the results show asymmetry, not corrected by trim-and-fill (Supplementary Figure S13).

Magnetic resonance imaging had the highest sensitivity (98%; 95% confidence interval [CI] 97–99%) while both CT scan and MRI had a high specificity (99%; 95% CI 96–100% and 99%; 95% CI 98–99%, respectively). In this meta-analysis, MRI had the best posterior probability of LR+ and LR− amongst other imaging modalities, with 96% and 100%, respectively, assuming a baseline 25% prior probability. Heterogeneity exists amongst all imaging modalities, although the heterogeneity is not statistically significant for MRI (I2 = 100 with a *p*-value of 0.5). Publication bias exists for both CT and MRI. According to the likelihood ratio scattergram, both CT scan and MRI can be used for exclusion and confirmation. Both imaging modalities have an excellent AUC with a very similar AUC value of 1 (0.99–1) (Supplementary Figure S14A,B). All of the imaging modalities have suggestive evidence (Table 2).

There are only two articles for ultrasonography and three for bone scan, hence they will be described qualitatively. In the ultrasound group, 124 patients were enrolled with 37 occult fractures [67,73]. Although both studies agree that ultrasonography is highly sensitive for occult hip and femoral fractures, with a sensitivity of 100% [73] and 96% (0.89–0.96) [67], the specificity differs wildly. Tsukamoto [67] found that ultrasound is highly specific with 0.98 (0.92–0.99), while Safran found a specificity of only 65% [73]. Nonetheless, both articles agree that ultrasonography can be used as an initial screening tool for patients with suspected occult hip and femoral fractures.

Three studies looked at the performance of bone scans in occult hip and femoral fractures. Out of all 139 patients enrolled across three studies, 50 had occult bone fractures. In these three studies, they agreed that MRI performs as well [76], if not better than bone scans [74,75]. These authors argue that MRI provides a faster scan (<15 min) and is well-tolerated by the patients, compared to delayed bone scans where not all centres provide imaging studies, and they are invasive and not cost-effective [74,75,76].

The model diagnostics are shown in Supplementary Figure S15A,B, where no studies appear to be outliers, as evidenced by the outlier detection for MRI, with a reasonable goodness-of-fit for MRI. However, one study seems to be an outlier for the CT scan study [83]. Deletion of this study resulted in a more stable model diagnostics, with a reasonable goodness-of-fit assumption for CT scans. However, there is not much change in the diagnostic accuracy of CT scans after the deletion of that study, with the same specificity and a sensitivity of 95%. Hence, the study was kept. The bivariate box plot showed a moderately homogenous performance of high sensitivity and specificity, providing indirect evidence of little to no threshold variability (Supplementary Figure S16A,B). Supplementary Figure S17A,B present the paired forest plot description of empirical Bayes predicted versus observed study-specific sensitivity and specificity, indicating threshold variability as sensitivity increases and specificity decreases and vice versa.

## 4. Discussion

This updated meta-analysis finds that the prevalence of occult hip and femoral fracture is higher than occult scaphoid fracture, with 44.8% as compared to 23.87%, respectively. The rate of occult hip and femoral fracture is not that far off from a previous systematic review with 39% (95% CI 35–43) [38]. The rate of occult scaphoid fracture is also similar to currently published studies, with a rate of around 20–25% [112]. In the past, this figure was as low as 5–10% [2]. The more prevalent uses of advanced imaging and increased awareness about occult fractures may have contributed to the rise of earlier detections. The higher risk of occult fracture in the hip and femur compared to occult scaphoid fracture may partly have to do with the population. Patients who suffer from occult hip and femoral fractures are usually older people, cachectic, suffer from malnutrition, or have other comorbidities that predispose them to falls, such as osteoporosis, Parkinson’s disease, dementia, or delirium [113,114]. Therefore, clinicians may find it difficult to ascertain with great confidence that an elderly individual, who may not fully cooperate, has a fracture when the initial radiographic result is negative. Meanwhile, those who suffer from occult fractures are usually younger, with a male predisposition, and with a clear history of trauma such as falling on outstretched hands, motor vehicle collision, or sports-related injuries [115]. 

Relying solely on sensitivity and specificity does not offer enough information to make informed decisions after receiving positive screening test results because of the occurrence of false positives [116]. There is also a trade-off between sensitivity and specificity, called the variability threshold, where if sensitivity rises, specificity falls, and vice versa [117]. Meanwhile, a limitation of utilizing the LR is its independence from prevalence, thereby impacting the accuracy of diagnostic tests. On the other hand, post-test probability, which is influenced by prevalence, emerges as a clinically more valuable parameter for assessing diagnostic test accuracy [118,119]. In this discussion, the focus will be heavily implied more on the posterior probability of LR+ and LR−.

In terms of post-test probability, MRI emerges as the best imaging modality to detect occult scaphoid and hip and femoral fractures. The next best imaging modality seems to be a CT scan for both fractures, but ultrasound has a better negative posterior probability than a CT scan. Our findings align with the ACR appropriateness criteria for both occult hip and scaphoid fractures, where MRI without IV contrast is typically employed as the first line and CT scan as an alternative [8,9]. In this analysis, the results show that the use of MRI, besides earlier detection of occult scaphoid fracture, can also exclude occult fractures. This finding is also supported by the ACR [8]. However, MRI cannot confidently exclude occult scaphoid fractures in this analysis, which is against the ACR document [8]. Some possibilities that may contribute to the discrepancies are the timing of imaging, the sampling methodologies, and the protocol, which will be discussed in-depth in the limitation section.

Despite numerous studies seemingly strengthening the argument for using earlier MRI to detect hip and scaphoid fractures, most centres are still reluctant to do so. One study finds that initial negative radiograph scaphoid fracture remains a persistent difficulty for the United Kingdom (UK) National Health Service (NHS), as numerous pathways demanding substantial resources rely on access to sophisticated imaging examinations. This difficulty is due to the constrained availability of advanced imaging in UK Emergency Departments for timely scanning of the scaphoid [120]. Numerous studies [5,121,122], including one randomized clinical trial for scaphoid fractures [123], have found that MRI is more cost-effective as compared to no further imaging or conventional follow-up radiographs for scaphoid fractures or CT scans for hip fractures.

Ultrasound in occult hip and femoral fractures is interesting as the previous systematic review excluded ultrasound as ultrasound was never used in clinical settings [39,124]. The primary radiographic signs often include scaphoid cortical disruption (a direct indication), radiocarpal fluid appearing hypoechoic due to hemarthrosis (with occasionally mixed echogenicity based on blood degradation stage), and effusion in the scaphoid–trapezium–trapezoid area (indirect indicators) [125]. The ACR appropriateness criteria also assign ultrasonography as “Usually Not Appropriate” for occult scaphoid fractures [8]. However, the assignment of this category is due to the lack of evidence, not due to evidence against its use [125]. Although it is inferior to MRI, its performance against CT scan as a second line is comparable, only falling short in the positive post-test probability department. Furthermore, ultrasound is more widely available, non-ionizing, and does not require special preparations (such as kidney function testing for contrast use in CT and MRI or sedation in paediatric patients undergoing MRI) [126]. Not all centres can afford CT scans or MRIs, and when they do, not all patients can afford them. Amongst all other imaging modalities for occult scaphoid fractures, ultrasound is the only diagnostic tool that can both exclude or confirm the fractures, with a low probability of publication bias. Although it is riddled with a high heterogeneity index, the use of ultrasound in occult scaphoid fracture merits further research.

The other imaging modalities mentioned in this analysis are CBCT and bone scan. While a bone scan is extremely sensitive, it is too costly and not appropriate for an emergency setting. Hence, it is not clinically viable and reflected in ACR appropriateness criteria for both occult hip and scaphoid fractures [8,9]. It is also worth mentioning that our analysis finds that bone scan has the lowest positive posterior probability and can neither confirm nor exclude occult scaphoid fractures. There is a low probability of heterogeneity and publication bias, which strengthens the arguments for reserving bone scans only in very special cases of occult scaphoid fractures. The use of CBCT is an emerging topic in the orthopaedics field. The main benefits compared to traditional CT lie in the fact that CBCT scanners enable quicker, more precise, quasi-three-dimensional imaging with significantly reduced radiation exposure, achieved through the utilization of smaller imaging devices [127]. It is also cheaper compared to CT scans [128]. One population-based, case-control study in France found that extremity CBCT in an emergency radiology department reduced the overall radiation dose, leading to an accelerated turnover, and it was feasible with a level of evidence of grade III [129]. The sentiment of the reduced dose is also expressed in another study, where they found that wrist CBCT involves a notably reduced scattered dose compared to wrist multi-sliced CT with similar diagnostic effectiveness. However, the use of CBCT entails a notably increased scattered radiation dosage to the neck, chest, and abdomen compared to scaphoid radiography from four views [130]. Similar to ultrasound, more studies are needed before the widespread clinical use of CBCT in occult fractures.

This umbrella review and updated meta-analysis suffers from several limitations. Firstly, there is a slight deviation in the protocol. Initially, the review aimed to include all occult fractures from all anatomical sites. However, the authors found that the scaphoid and hip are the two most extensive anatomical sites for occult fractures being studied, with no other systematic reviews on other anatomical sites. There is one systematic review on occult ankle fractures in the paediatric population, but further scrutiny found that not all included fractures were occult fractures [131]. Secondly, publications on some imaging modalities suffer from heterogeneity and publication bias. An attempt was made to meta-regress the findings to possibly elucidate the source of heterogeneity. However, several substantial missing data from the original publications made it difficult to uncover the sources of heterogeneity. Some sources of heterogeneity may include study methodology, the clinical context of the study (in-clinic vs. emergency department), study protocol (the reference test of choice and when the next follow-up is), the pre-test probability of each patient having occult fractures, the imaging protocols, and the number, experiences, and expertise of the interpreters. The fourth limitation is that most of the included studies suffer from some risk of bias, which may impede the clinical implementation of these imaging modalities in diagnosing occult fractures. The fifth limitation lies in the fact that the results of CBCT for occult scaphoid fractures and ultrasonography and bone scans for occult hip and femoral fractures cannot be synthesized into a meta-analysis, unlike the previous meta-analysis [38]. However, this limitation also underlines our strengths in reviewing every single manuscript for the inclusion and exclusion criteria. Despite being more time-consuming, our meta-analyses found that many primary articles that were previously included under very similar inclusion and exclusion criteria ended up not fulfilling the criteria. Lastly, despite a very thorough search, the stratification of evidence is still weak for occult scaphoid fractures and only suggestive for occult hip and femoral fractures.

## 5. Conclusions

The quest to find the best imaging modality for occult fractures has not ended here. Despite the limitations, our umbrella review and meta-analysis encompass the most recent literature around occult scaphoid and hip and femoral fractures. The findings strengthen the case for use of MRI after initially negative radiograph fracture for occult hip and femoral fractures, with a CT scan as a viable second option. However, the evidence is only suggestive at best. As for occult scaphoid fractures, MRI is still the first-line imaging choice with CT scan being the second option and ultrasonography being a potential candidate that needs further studies. Bone scans should be out of favour in detecting occult fractures and it may be worthwhile to conduct further studies on ultrasound in the detection of occult fractures. However, overall weak evidence for all imaging modalities of occult scaphoid fractures means that more evidence is needed.

The adoption of multimodality imaging into the guidelines does not ensure clinical translation. Although further large, prospective, multi-centre studies are welcomed, studies that explore the clinical inertia of using MRI as a first-line imaging modality to occult fractures or studies that explore the challenges faced by hospitals, clinicians, and their workload if all patients with occult fractures underwent MRI are also urgently needed. There is also a need for analysis that investigates the adverse impact of overdiagnosis when MRI is routinely implemented [132]. Lastly, although it seems intuitive that other modalities such as fludeoxyglucose (FDG)–positron emission tomography (PET) scan [133,134], PET/CT, PET/MRI scan [135], and single-photon emission computed tomography (SPECT) [136] may not be used for occult fractures, studies will still need to be done to determine definitively that these modalities are not needed in occult fractures or other indications and justifications for their use.

## Figures and Tables

**Table 1:** Characteristics of systematic reviews and meta-analysis included in the umbrella review.

Author (Year)	Objectives	Anatomy	Setting and Context	Number of Databases Sourced and Searched	Date Range of Database Searching	Publication Date Range of Studies Included in the Review that Inform Each Outcome of Interest	Number of Studies	Types of Studies	Total Number of Patients	Country of Origin of Studies	Instruments Used to Appraise the Primary Studies	AMSTAR	Relevant Outcomes Reported	Methods of Synthesis	Publication Bias	Heterogeneity	Risk of Bias
Chatha (2011) [35]	To determine the role of CT and MRI in diagnosing occult fracture	Femur	N/A	5 (MEDLINE, EMBASE, CINAHL, Google Scholar, and the Cochrane Library)	31 October 2009	1989 to 2009	22	15 prospective and 7 retrospective	996	N/A	None	Critically low	MRI was an investigation of choice for occult proximal femoral fractures.	Qualitative	N/A	N/A	N/A
Kellock (2019) [36]	The diagnostic performance of CT for the detection of occult proximal femoral fracture	Femur	N/A	3 (Pubmed, Embase, and Web of Science)	1 December 2018	2005 to 2018	13	1 prospective, the rest are retrospective	1248	5 UK, 1 USA, 1 Israel, 3 Sweden, 1 Romania, 1 Denmark, 1 Canada	QUADAS-2	High Quality	The estimated summary sensitivity of CT for the detection of nondisplaced hip fracture was 94% (95% credible interval, 83–99%), and the specificity was 100% (95% credible interval, 99–100%) with an AUC of 0.981.	Meta-analysis	Present	Present	High risk of bias in the reference standard, with no single study achieving a good quality
Wilson (2019) [37]	Evaluate the diagnostic accuracy of limited MRI protocols for detecting radiographically occult proximal femoral fractures	Femur	All single-centre, academic hospitals	4 (MEDLINE, Embase, Cochrane Library and Scopus)	15 November 2019	1993–2019 (2012 to 2019 for meta-analysis; 1993 to 2016 for systematic review)	11 (only five for meta-analysis)	5 Prospective, 6 Retrospective (1 prospective and 4 retrospective for meta-analysis; 4 prospective and 2 retrospective for systematic review)	938	N/A	QUADAS-2	High Quality	The pooled and weighted summary sensitivity and specificity and the area under the summary ROC curve for limited MRI protocols in detecting radiographically occult hip fractures were 99% (95% CI, 91–100%), 99% (95% CI, 97–100%), and 100% (95% CI, 0.99–1), respectively.	Qualitative and Meta-Analysis	N/A	N/A	All studies included in the meta-analysis were low-risk
Haj-Mirzaian (2020) [38]	Determine the diagnostic performance of CT and bone scanning in the detection of occult fractures using MRI as the reference standard	Femoral head, femoral neck, intertrochanteric, or subtrochanteric fractures	N/A	3 (Pubmed, EMBASE, and Cochrane Library)	27 September 2018	1993 to 2018	35	N/A	2992	N/A	ROB	High Quality	CT and bone scanning yielded comparable diagnostic performance in the detection of radiographically occult hip fracture (*p* = 0.67) with a sensitivity of 79% and 87%, respectively.	Meta-analysis	Present	Present	Low ROB = 27, Moderate ROB = 6, High ROB = 2
Mallee (2015) [39]	identify the most suitable diagnostic imaging strategy for identifying clinically suspected fractures of the scaphoid bone in patients with normal radiographs	Scaphoid	People of all ages who presented at the hospital or clinics within one week of trauma with a clinically suspected scaphoid fracture and negative post-trauma radiographs	10 (Cochrane Register of Diagnostic Test Accuracy Studies, MEDLINE, EMBASE, the Database of Abstracts of Reviews of EHects, the Cochrane Central Register of Controlled Trials, the NHS Economic Evaluation Database, MEDION, ARIF, Current Controlled Trials, the World Health Organization (WHO) International Clinical Trials Registry Platform)	July 2012	1983–2011	11	9 Prospective, 3 Not Reported	1041	5 Netherlands, 2 Austria, 1 Turkey, 1 Denmark, 1 Ireland, 1 Norway	Not Specified	High Quality	Summary sensitivity and specificity of CT were 0.72 (95% confidence interval (CI) 0.36 to 0.92) and 0.99 (95% CI 0.71 to 1.00); for MRI, these were 0.88 (95% CI 0.64 to 0.97) and 1.00 (95% CI 0.38 to 1.00); for BS, these were 0.99 (95% CI 0.69 to 1.00) and 0.86 (95% CI 0.73 to 0.94). Indirect comparisons suggest that the diagnostic accuracy of BS was significantly higher than CT and MRI, and CT and MRI have comparable diagnostic accuracy.	Meta-analysis	Present	Could not be investigated formally	Five studies were considered “good quality”, and six studies had “moderate quality”.
Kwee (2018) [40]	To systematically review the literature on the performance of ultrasound in diagnosing radiographically occult scaphoid fracture.	Scaphoid	N/A	2 (MEDLINE and Embase databases)	No Limit–8 January 2018	2001–2013	7	Not Specified	314	1 Turkey, 2 Switzerland, 1 USA, 1 France, 1 Austria, 1 UK	QUADAS-2	Critically low	The sensitivity and specificity of ultrasound in diagnosing radiographically occult scaphoid fracture ranged from 77.8% to 100% and from 71.4% to 100% respectively, with pooled estimates of 85.6% (95% CI: 73.9%, 92.6%) and 83.3% (95% CI: 72.0%, 90.6%), respectively.	Meta-analysis	Not Assessed	Cannot be explored	Index Test: Low ROB: 6, High ROB: 1; Reference Standard: High ROB: 2, Unclear: 2; Low ROB: 3; Patient Selection and Flow and Timing: Low ROB 7.

**Table 2:** Pooled estimates of diagnostic performance of multimodality imaging for diagnosis of radiographically occult hip and femur and scaphoid fractures.

Parameter	Scaphoid				Hip and Femur	
	Ultrasound	CT Scan	MRI	Bone Scan	CT Scan	MRI
No. of studies	6	5	8	7	15	29
No. of patients	270	297	345	586	1329	1905
Sensitivity (%)	96 (66–100)	81 (64–91)	86 (68–94)	98 (90–100)	94 (80–99)	98 (97–99)
Specificity (%)	94 (66–99)	98 (86–100)	98 (28–100)	80 (44–95)	99 (96–100)	99 (98–99)
Positive likelihood ratio	16 (2.2–114.4)	41.7 (5.5–316.6)	48.7 (0.4–6437.6)	4.9 (1.3–17.9)	82.1 (22.6–298.5)	69 (41–116.1)
Posterior probability (%) assuming a 25% prior probability	84	93	95	62	96	96
Negative likelihood ratio	0.04 (0–0.47)	0.2 (0.1–0.39)	0.15 (0.06–0.35)	0.02 (0–0.16)	0.06 (0.01–0.22)	0.02 (0.01–0.03)
Posterior probability (%) assuming a 25% prior probability	1	6	0.15	1	2	1
Positive predictive value	0.95 (0.9–1)	0.82 (0.8–0.84)	0.86 (0.84–0.88)	0.97 (0.85–1)	82.1 (22.6–298.5)	69 (41–116.1)
Negative predictive value	0.93 (0.88–0.99)	0.97 (0.95–1)	0.98 (0.95–1)	0.81 (0.7–0.92)	0.06 (0.01–0.22)	0.02 (0.01–0.03)
Diagnostic odds ratio	387 (17–8879)	210 (31–1449)	334 (2–49,601)	213 (12–3743)	1425 (171–11851)	4185 (2009–8720)
Area under the curve	0.99 (0.2–1)	0.91 (0.17–1)	0.91 (0.89–0.94)	0.99 (0.97–0.99)	1 (0.99–1)	1 (0.99–1)
I^2^ (%) and *p*-value	81 (59–100) and 0.003	85 (70–100) and 0.001	96 (93–99) and <0.0001	1 (0–100) and 0.182	92 (83–100) and <0.0001	100 (0–100) and 0.5
Publication bias	0.56	0.03	0.07	0.13	<0.001	<0.001
Likelihood ratio scattergram	LUQ; Exclusion and confirmation	RUQ; Confirmation only	RUQ; Confirmation only	RLQ; No exclusion or confirmation	LUQ; Exclusion and confirmation	LUQ; Exclusion and confirmation
Stratification of evidence	Weak	Weak	Weak	Weak	Suggestive	Suggestive

CT, computed tomography; MRI, magnetic resonance imaging; LUQ, left upper quadrant; RUQ, right upper quadrant; RLQ, right lower quadrant.

## Data Availability

Available upon request.

## Supplementary Materials

The following supporting information can be downloaded at: https://www.mdpi.com/article/10.3390/jcm13133769/s1, File S1: PRISMA 2020 Checklist; Table S1: Medical subject heading (MeSH) terms used in each database for the umbrella study, Table S2: Medical subject heading (MeSH) terms used in each database for the hip or femoral fractures, Table S3: Medical subject heading (MeSH) terms used in each database for the scaphoid fractures, Table S4: Notable exclusions for the umbrella review, Table S5: Notable exclusions for the scaphoid fracture, Table S6: Machines and protocols used, Table S7: Descriptive characteristics of each study included in occult scaphoid fracture, Table S8: Notable exclusions for the hip fracture, Figure S1: PRISMA flowchart for selection of included studies in the umbrella review, Figure S2: PRISMA flowchart for selection of included studies in the systematic review of the occult fractures of the scaphoid, Figure S3: Meta-analysis of prevalence of occult scaphoid fracture, Figure S4: Galbraith plot of occult scaphoid fracture, Figure S5: Funnel plot of studies included in the prevalence of occult scaphoid fracture, Figure S6: The hierarchical summary receiver operating characteristic (HSROC) of ultrasound (A), CT (B), MRI (C), and bone scan (D) in detecting occult scaphoid fracture, Figure S7: Model diagnostics of each study for ultrasound (A), CT (B), MRI (C), and bone scan (D) in detecting occult scaphoid fracture, Figure S8: Bivariate boxplot of each study for ultrasound (A), CT (B), MRI (C), and bone scan (D) in detecting occult scaphoid fracture, Figure S9: Empirical Bayes prediction of sensitivity and specificity of each study for ultrasound (A), CT (B), MRI (C), and bone scan (D) in detecting occult scaphoid fracture, Figure S10: PRISMA flowchart for selection of included studies in the systematic review of the occult fractures of the hip, Figure S11: Meta-analysis of prevalence of occult hip and femoral fracture, Figure S12: Galbraith plot of occult scaphoid fracture, Figure S13: Funnel plot of studies included in the prevalence of occult hip and femoral fracture, Figure S14: The hierarchical summary receiver operating characteristic (HSROC) of CT (A) and MRI (B) in detecting occult hip and femoral fracture, Figure S15: Model diagnostics of each study for CT (A) and MRI (B) in detecting occult hip and femoral fracture, Figure S16: Model diagnostics of each study for CT (A) and MRI (B) in detecting occult hip and femoral fracture, Figure S17: Empirical Bayes prediction of sensitivity and specificity of each study for CT (A) and MRI (B) in detecting occult hip and femoral fracture.

## Appendix A

**Table A1:** QUADAS-2 graphical representation of the risk-of-bias and concerns regarding applicability of occult scaphoid fractures.

Study	Risk of Bias				Applicability Concerns			Conclusions
	Patient Selection	Index Test	Reference Standard	Flow and Timing	Patient Selection	Index Test	Reference Standard	
Yildirim (2013) [46]	☺	☺	?	☺	☺	☺	☺	At risk of bias
Platon (2011) [47]	☺	☺	☺	☺	☺	☺	☺	Low risk of bias
Fusetti (2005) [48]	☺	☺	☺	☺	☺	☺	☺	Low risk of bias
Senall (2004) [49]	☺	☺	☹	☺	☺	☺	☺	At risk of bias
Hauger (2002) [50]	☺	☺	☺	☺	☺	☺	☺	Low risk of bias
Herneth (2001) [51]	☺	?	?	☺	☺	☺	☺	At risk of bias
Xie (2020) [41]	☹	?	☺	☺	?	?	☺	At risk of bias
Kitsis (1989) [42]	☹	?	?	☺	☺	☺	☺	At risk of bias
Thorpe (1996) [43]	☺	?	?	☺	☺	☺	☺	At risk of bias
Fowler (1998) [44]	☺	?	☺	☺	☺	?	☺	At risk of bias
Borel (2017) [63]	☺	?	☺	☺	☺	☺	☺	At risk of bias
Edlund (2016) [64]	☺	☺	☺	☹	☺	☺	☺	At risk of bias
De Zwart (2012) [52]	?	☹	☹	☺	☺	☹	☺	At risk of bias
Beeres (2008) [56]	☺	☹	☹	☺	☺	☹	☺	At risk of bias
Ilica (2011) [53]	☹	☺	☺	☹	☺	☺	☺	At risk of bias
Mallee (2011) [54]	☺	?	☺	☺	☺	☺	☺	At risk of bias
Memasadeghi (2006) [55]	?	☺	?	☺	☺	☺	☺	At risk of bias
Breitenseher (1997) [57]	☺	☺	☺	☺	☺	☺	☺	Low risk of bias
Tiel-van Buul (1996) [58]	?	?	?	☺	☺	☺	☺	At risk of bias
Nielsen (1983) [62]	?	?	?	☺	☺	☺	☺	At risk of bias
O’Carroll (1982) [61]	☹	☹	☹	☹	☹	☹	☹	At risk of bias
Stordahl (1984) [60]	?	☺	?	?	?	☺	?	At risk of bias
Tiel-van Buul (1993) [59]	☺	☺	?	☺	☺	☺	☺	At risk of bias

☺ Low Risk; ☹ High Risk; ? Unclear Risk.

**Table A2:** QUADAS-2 graphical representation of the risk-of-bias and concerns regarding applicability of occult hip and femoral fractures.

Study	Risk of Bias				Applicability Concerns			Conclusions
	Patient Selection	Index Test	Reference Standard	Flow and Timing	Patient Selection	Index Test	Reference Standard	
Deutsch (1989) [89]	☹	☹	?	☺	?	?	?	At risk of bias
Rizzo (1993) [76]	☹	☹	?	☺	?	?	?	At risk of bias
Quinn and McCarthy (1993) [90]	☺	☹	☹	☺	☺	☹	☺	At risk of bias
Evans (1994) [75]	☺	☹	?	☺	?	?	?	At risk of bias
Haramati (1994) [91]	☹	☹	☹	☹	☹	☹	☹	At risk of bias
Bogost (1995) [92]	?	☹	☹	☺	?	☺	☺	At risk of bias
Stiris and Lilleas (1997) [93]	?	☹	☹	☺	?	☺	☺	At risk of bias
Rubin (1998) [74]	?	?	?	☺	☺	☺	☺	At risk of bias
Pandey (1998) [94]	☹	☹	☹	☹	☹	☹	☹	At risk of bias
Lim (2002) [95]	☺	☹	☹	☺	☺	☹	☹	At risk of bias
Oka and Monu (2004) [96]	☺	☹	☹	☺	☺	☹	☹	At risk of bias
Galloway (2004) [97]	☹	?	☺	?	☺	☺	☺	At risk of bias
Lee (2004) [98]	☹	?	☺	?	☺	☺	☺	At risk of bias
Alam (2005) [99]	☺	?	☺	☺	☺	☺	☺	At risk of bias
Frihagen (2005) [100]	☺	☹	☹	☺	☺	☹	☹	At risk of bias
Verbeeten (2005) [101]	☺	?	☺	☺	☺	☺	☺	At risk of bias
Lubovsky (2005) [88]	☹	?	☺	☹	☹	?	☺	At risk of bias
Dominguez (2005) [102]	☹	?	☺	☺	?	☺	☺	At risk of bias
Chana (2006) [103]	?	☹	☺	?	☺	☺	?	At risk of bias
Hossain (2007) [104]	?	?	☺	☺	☺	☺	☺	At risk of bias
Sankey (2009) [105]	☺	☹	☺	☺	☺	☺	☺	At risk of bias
Safran (2009) [73]	☺	☺	☺	☺	☺	☺	☺	Low risk of bias
Szewczyk (2012) [106]	☺	☺	☺	☺	☺	☺	☺	Low risk of bias
Iwata (2012) [107]	☺	?	☺	☺	☺	?	☺	At risk of bias
Dunker (2012) [80]	☺	☺	☹	☺	☺	☺	☹	At risk of bias
Ohishi (2012) [108]	☺	☺	☺	☺	☺	☺	☺	Low risk of bias
Geijer (2012) [79]	☺	☺	☹	?	☺	☺	☹	At risk of bias
Gill (2013) [77]	☹	☺	☹	?	☹	?	☹	At risk of bias
Heikal (2014) [81]	☺	☺	☹	☹	☺	☺	☹	At risk of bias
Haubro (2015) [82]	☺	☺	☺	☺	☺	☺	☺	Low risk of bias
Deleanu (2015) [83]	☺	?	☹	☹	☹	?	☹	At risk of bias
Collin (2016) [72]	☺	☺	☺	☺	☺	☺	☺	Low risk of bias
Rehman (2016) [78]	☺	?	☹	?	☺	?	☹	At risk of bias
Sadozai (2016) [84]	☺	☺	☹	?	☺	☺	☹	At risk of bias
Thomas (2016) [85]	☹	☺	☹	?	☺	☺	☹	At risk of bias
Lakshmanan (2017) [109]	☺	?	☺	☺	☺	?	☺	At risk of bias
Lord (2017) [110]	☺	☺	☺	☺	☺	☺	☺	Low risk of bias
Mandell (2018) [86]	☺	☺	☹	☺	☺	☺	☹	At risk of bias
Ross (2019) [111]	☺	☺	☺	☺	☺	☺	☺	Low risk of bias
Heynen (2019) [87]	☺	☺	☺	☺	☺	☺	☺	Low risk of bias
Haims (2020) [66]	☹	?	?	☺	☹	?	☺	At risk of bias
Lanotte (2019) [65]	☹	☺	?	☹	☹	☺	☺	At risk of bias
Kutaiba (2020) [68]	☹	☺	☺	?	?	?	☺	At risk of bias
Tsukamoto (2023) [67]	☹	☹	?	?	?	?	?	At risk of bias
Reddy (2015) [69]	☹	☺	☹	?	☹	?	☹	At risk of bias

☺ Low Risk; ☹ High Risk; ? Unclear Risk.
