# Supplementary material for: An Umbrella Review and Updated Meta-Analysis of Imaging Modalities in Occult Scaphoid and Hip and Femoral Fractures
Source: J Clin Med. 2024 Jun 27;13(13):3769. doi: 10.3390/jcm13133769 (PMC11242027; doi:10.3390/jcm13133769)
Supplement: Supplementary file 1 [file jcm-13-03769-s001.zip › Supplementary Figures and Tables Occult Fracture.pdf]

Supplementary Table S1. Medical subject heading (MeSH) terms used in each database for the umbrella study

| Database | Medical Subject Heading | Number of studies found |
|---|---|---|
| Pubmed | (("fractures, closed"[MeSH Terms] OR ("fractures"[All Fields] AND "closed"[All Fields]) OR "closed fractures"[All Fields] OR ("occult"[All Fields] AND "fracture"[All Fields]) OR "occult fracture"[All Fields] OR (("suspect"[All Fields] OR "suspected"[All Fields] OR "suspecting"[All Fields] OR "suspects"[All Fields]) AND ("fractur"[All Fields] OR "fractural"[All Fields] OR "fracture s"[All Fields] OR "fractures, bone"[MeSH Terms] OR ("fractures"[All Fields] AND "bone"[All Fields]) OR "bone fractures"[All Fields] OR "fracture"[All Fields] OR "fractured"[All Fields] OR "fractures"[All Fields] OR "fracturing"[All Fields])) OR (("negative"[All Fields] OR "negatively"[All Fields] OR "negatives"[All Fields] OR "negativities"[All Fields] OR "negativity"[All Fields]) AND ("radiograph"[All Fields] OR "radiographed"[All Fields] OR "radiographer"[All Fields] OR "radiographer s"[All Fields] OR "radiographers"[All Fields] OR "radiographic"[All Fields] OR "radiographical"[All Fields] OR "radiographically"[All Fields] OR "radiographics"[All Fields] OR "radiographing"[All Fields] OR "radiographs"[All Fields]) AND ("fractur"[All Fields] OR "fractural"[All Fields] OR "fracture s"[All Fields] OR "fractures, bone"[MeSH Terms] OR ("fractures"[All Fields] AND "bone"[All Fields]) OR "bone fractures"[All Fields] OR "fracture"[All Fields] OR "fractured"[All Fields] OR "fractures"[All Fields] OR "fracturing"[All Fields])) OR ("non-displaced"[All Fields] AND ("fractur"[All Fields] OR "fractural"[All Fields] OR "fracture s"[All Fields] OR "fractures, bone"[MeSH Terms] OR ("fractures"[All Fields] AND "bone"[All Fields]) OR "bone fractures"[All Fields] OR "fracture"[All Fields] OR "fractured"[All Fields] OR "fractures"[All Fields] OR "fracturing"[All Fields]))) AND ("tomography, x ray computed"[MeSH Terms] OR ("tomography"[All Fields] AND "x ray"[All Fields] AND "computed"[All Fields]) OR "x-ray computed tomography"[All Fields] OR ("computed"[All Fields] AND "tomography"[All Fields]) OR "computed tomography"[All Fields] OR ("magnetic resonance imaging"[MeSH Terms] OR ("magnetic"[All Fields] AND "resonance"[All Fields] AND "imaging"[All Fields]) OR "magnetic resonance imaging"[All Fields]) OR ("cone beam computed tomography"[MeSH Terms] OR ("cone beam"[All Fields] AND "computed"[All Fields] AND "tomography"[All Fields]) OR "cone beam computed tomography"[All Fields] OR ("cone"[All Fields] AND "beam"[All Fields] AND "computed"[All Fields] AND "tomography"[All Fields]) OR "cone beam computed tomography"[All Fields]) OR ("diagnostic imaging"[MeSH Subheading] OR ("diagnostic"[All Fields] AND "imaging"[All Fields]) OR "diagnostic imaging"[All Fields] OR "x ray"[All Fields] OR "x rays"[MeSH Terms] OR "x rays"[All Fields]) OR (("bone and bones"[MeSH Terms] OR ("bone"[All Fields] AND "bones"[All Fields]) OR "bone and bones"[All Fields] OR "bone"[All Fields]) AND ("radionuclide imaging"[MeSH Terms] OR ("radionuclide"[All Fields] AND "imaging"[All Fields]) OR "radionuclide imaging"[All Fields] OR "scan"[All Fields])) OR "tomosynthesis"[All Fields] OR ("diagnostic imaging"[MeSH Subheading] OR ("diagnostic"[All Fields] AND "imaging"[All Fields]) OR "diagnostic imaging"[All Fields] OR "ultrasonography"[All Fields] OR "ultrasonography"[MeSH Terms] OR "ultrasonographies"[All Fields]))) AND (meta-analysis[Filter] OR systematicreview[Filter]) | 95 |
| Medline | ((((((occult fracture[MeSH Terms]) OR suspected fracture) OR negative radiograph fracture) OR non-displaced fracture)) AND ((systematic review[Title]) OR meta-analysis[Title])) AND ((((((((computed tomography[MeSH Terms]) OR magnetic resonance imaging[MeSH Terms]) OR cone beam computed tomography[MeSH Terms]) OR x-ray[MeSH Terms]) OR bone scan) OR tomosynthesis)) OR (ultrasonography[MeSH Terms])) | 267 |
| Cochrane Library | (fractures):ti,ab,kw AND ("X ray"):ti,ab,kw OR ("computed tomography scan"):ti,ab,kw OR ("magnetic resonance imaging scan"):ti,ab,kw OR ("bone scan index"):ti,ab,kw | 16 |
| Google Scholar | fracture diagnostic "Systematic review" -dental -deep -learning -management -treatment -outcome | 5,610 |
| Science Direct | fracture AND (Systematic review) AND (Diagnostic) filter review article | 6,500 |

Supplementary Table S2. Medical subject heading (MeSH) terms used in each database for the hip or femoral fractures

| Database | Medical Subject Heading | Number of studies found |
|---|---|---|
| Pubmed | (("hip"[MeSH Terms] OR "hip"[All Fields] OR ("femur"[MeSH Terms] OR "femur"[All Fields] OR "femurs"[All Fields] OR "femur s"[All Fields] OR "femural"[All Fields] OR "femure"[All Fields]) OR (("proximal"[All Fields] OR "proximalization"[All Fields] OR "proximalize"[All Fields] OR "proximalized"[All Fields] OR "proximalizes"[All Fields] OR "proximalizing"[All Fields] OR "proximally"[All Fields] OR "proximals"[All Fields]) AND ("fractur"[All Fields] OR "fractural"[All Fields] OR "fracture s"[All Fields] OR "fractures, bone"[MeSH Terms] OR ("fractures"[All Fields] AND "bone"[All Fields]) OR "bone fractures"[All Fields] OR "fracture"[All Fields] OR "fractured"[All Fields] OR "fractures"[All Fields] OR "fracturing"[All Fields]))) AND ("tomography, x ray computed"[MeSH Terms] OR ("tomography"[All Fields] AND "x ray"[All Fields] AND "computed"[All Fields]) OR "x-ray computed tomography"[All Fields] OR ("computed"[All Fields] AND "tomography"[All Fields]) OR "computed tomography"[All Fields] OR ("magnetic resonance imaging"[MeSH Terms] OR ("magnetic"[All Fields] AND "resonance"[All Fields] AND "imaging"[All Fields]) OR "magnetic resonance imaging"[All Fields]) OR ("cone beam computed tomography"[MeSH Terms] OR ("cone beam"[All Fields] AND "computed"[All Fields] AND "tomography"[All Fields]) OR "cone beam computed tomography"[All Fields] OR ("cone"[All Fields] AND "beam"[All Fields] AND "computed"[All Fields] AND "tomography"[All Fields]) OR "cone beam computed tomography"[All Fields]) OR (("bone and bones"[MeSH Terms] OR ("bone"[All Fields] AND "bones"[All Fields]) OR "bone and bones"[All Fields] OR "bone"[All Fields]) AND ("radionuclide imaging"[MeSH Terms] OR ("radionuclide"[All Fields] AND "imaging"[All Fields]) OR "radionuclide imaging"[All Fields] OR "scan"[All Fields])) OR ("diagnostic imaging"[MeSH Subheading] OR ("diagnostic"[All Fields] AND "imaging"[All Fields]) OR "diagnostic imaging"[All Fields] OR "ultrasonography"[All Fields] OR "ultrasonography"[MeSH Terms] OR "ultrasonographies"[All Fields])) AND ("fractures, closed"[MeSH Terms] OR ("fractures"[All Fields] AND "closed"[All Fields]) OR "closed fractures"[All Fields] OR ("occult"[All Fields] AND "fracture"[All Fields]) OR "occult fracture"[All Fields] OR (("negative"[All Fields] OR "negatively"[All Fields] OR "negatives"[All Fields] OR "negativities"[All Fields] OR "negativity"[All Fields]) AND ("radiograph"[All Fields] OR "radiographed"[All Fields] OR "radiographer"[All Fields] OR "radiographer s"[All Fields] OR "radiographers"[All Fields] OR "radiographic"[All Fields] OR "radiographical"[All Fields] OR "radiographically"[All Fields] OR "radiographics"[All Fields] OR "radiographing"[All Fields] OR "radiographs"[All Fields]) AND ("fractur"[All Fields] OR "fractural"[All Fields] OR "fracture s"[All Fields] OR "fractures, bone"[MeSH Terms] OR ("fractures"[All Fields] AND "bone"[All Fields]) OR "bone fractures"[All Fields] OR "fracture"[All Fields] OR "fractured"[All Fields] OR "fractures"[All Fields] OR "fracturing"[All Fields])) OR ("nondisplaced"[All Fields] AND ("fractur"[All Fields] OR "fractural"[All Fields] OR "fracture s"[All Fields] OR "fractures, bone"[MeSH Terms] OR ("fractures"[All Fields] AND "bone"[All Fields]) OR "bone fractures"[All Fields] OR "fracture"[All Fields] OR "fractured"[All Fields] OR "fractures"[All Fields] OR "fracturing"[All Fields])))) AND (2018:2024[pdat]) | 502 |
| Medline | [((Occult fracture) OR negative radiograph fracture) OR non-displaced fracture] AND ((Hip[MeSH Terms]) OR femur[MeSH Terms]) OR proximal femur[MeSH Terms] AND (((((computed tomography[MeSH Terms]) OR magnetic resonance imaging[MeSH Terms]) OR cone beam computed tomography[MeSH Terms]) OR bone scan[MeSH Terms]) OR ultrasonography[MeSH Terms]) AND ("2018/01/01"[PubDate] : "2024/03/25"[PubDate]) | 108 |
| Cochrane Library | (fractures):ti,ab,kw AND ("X ray"):ti,ab,kw OR ("computed tomography scan"):ti,ab,kw OR ("magnetic resonance imaging scan"):ti,ab,kw OR ("bone scan index"):ti,ab,kw AND (hip):ti,ab,kw | 2 |
| Google Scholar | Hip OR Femur "occult fracture" -marrow (from 2018-2024) | 820 |
| Science Direct | (Occult Fracture OR Nondisplaced Fracture) AND (CT Scan OR MRI OR Ultrasonography OR CBCT) AND (Femur OR Hip) from 2018-2024 and research articles only | 291 |

Supplementary Table S3. Medical subject heading (MeSH) terms used in each database for the scaphoid fractures

| Database | Medical Subject Heading | Number of studies found |
|---|---|---|
| Pubmed | "scaphoid bone"[MeSH Terms] OR ("scaphoid"[All Fields] AND "bone"[All Fields]) OR "scaphoid bone"[All Fields] OR "scaphoid"[All Fields] OR "scaphoid s"[All Fields] OR "scaphoids"[All Fields]AND ("fractur"[All Fields] OR "fractural"[All Fields] OR "fracture s"[All Fields] OR "fractures, bone"[MeSH Terms] OR ("fractures"[All Fields] AND "bone"[All Fields]) OR "bone fractures"[All Fields] OR "fracture"[All Fields] OR "fractured"[All Fields] OR "fractures"[All Fields] OR "fracturing"[All Fields]))) AND ("tomography, x ray computed"[MeSH Terms] OR ("tomography"[All Fields] AND "x ray"[All Fields] AND "computed"[All Fields]) OR "x-ray computed tomography"[All Fields] OR ("computed"[All Fields] AND "tomography"[All Fields]) OR "computed tomography"[All Fields] OR ("magnetic resonance imaging"[MeSH Terms] OR ("magnetic"[All Fields] AND "resonance"[All Fields] AND "imaging"[All Fields]) OR "magnetic resonance imaging"[All Fields]) OR ("cone beam computed tomography"[MeSH Terms] OR ("cone beam"[All Fields] AND "computed"[All Fields] AND "tomography"[All Fields]) OR "cone beam computed tomography"[All Fields] OR ("cone"[All Fields] AND "beam"[All Fields] AND "computed"[All Fields] AND "tomography"[All Fields]) OR "cone beam computed tomography"[All Fields]) OR (("bone and bones"[MeSH Terms] OR ("bone"[All Fields] AND "bones"[All Fields]) OR "bone and bones"[All Fields] OR "bone"[All Fields]) AND ("radionuclide imaging"[MeSH Terms] OR ("radionuclide"[All Fields] AND "imaging"[All Fields]) OR "radionuclide imaging"[All Fields] OR "scan"[All Fields])) OR ("diagnostic imaging"[MeSH Subheading] OR ("diagnostic"[All Fields] AND "imaging"[All Fields]) OR "diagnostic imaging"[All Fields] OR "ultrasonography"[All Fields] OR "ultrasonography"[MeSH Terms] OR "ultrasonographies"[All Fields])) AND ("fractures, closed"[MeSH Terms] OR ("fractures"[All Fields] AND "closed"[All Fields]) OR "closed fractures"[All Fields] OR ("occult"[All Fields] AND "fracture"[All Fields]) OR "occult fracture"[All Fields] OR (("negative"[All Fields] OR "negatively"[All Fields] OR "negatives"[All Fields] OR "negativities"[All Fields] OR "negativity"[All Fields]) AND ("radiograph"[All Fields] OR "radiographed"[All Fields] OR "radiographer"[All Fields] OR "radiographer s"[All Fields] OR "radiographers"[All Fields] OR "radiographic"[All Fields] OR "radiographical"[All Fields] OR "radiographically"[All Fields] OR "radiographics"[All Fields] OR "radiographing"[All Fields] OR "radiographs"[All Fields]) AND ("fractur"[All Fields] OR "fractural"[All Fields] OR "fracture s"[All Fields] OR "fractures, bone"[MeSH Terms] OR ("fractures"[All Fields] AND "bone"[All Fields]) OR "bone fractures"[All Fields] OR "fracture"[All Fields] OR "fractured"[All Fields] OR "fractures"[All Fields] OR "fracturing"[All Fields])) OR ("nondisplaced"[All Fields] AND ("fractur"[All Fields] OR "fractural"[All Fields] OR "fracture s"[All Fields] OR "fractures, bone"[MeSH Terms] OR ("fractures"[All Fields] AND "bone"[All Fields]) OR "bone fractures"[All Fields] OR "fracture"[All Fields] OR "fractured"[All Fields] OR "fractures"[All Fields] OR "fracturing"[All Fields])))) AND (2020:2024[pdat]) | 193 |
| Medline | [((Occult fracture) OR negative radiograph fracture) OR non-displaced fracture] AND ((Scaphoid[MeSH Terms])) AND (((((computed tomography[MeSH Terms]) OR magnetic resonance imaging[MeSH Terms]) OR cone beam computed tomography[MeSH Terms]) OR bone scan[MeSH Terms]) OR ultrasonography[MeSH Terms]) AND ("2020/01/01"[PubDate] : "2024/03/25"[PubDate]) | 5 |
| Cochrane Library | (fractures):ti,ab,kw AND ("X ray"):ti,ab,kw OR ("computed tomography scan"):ti,ab,kw OR ("magnetic resonance imaging scan"):ti,ab,kw OR ("bone scan index"):ti,ab,kw AND (scaphoid):ti,ab,kw | 1 |
| Google Scholar | Scaphoid "occult fracture" -marrow (from 2020-2024) | 14 |
| Science Direct | (Occult Fracture OR Nondisplaced Fracture) AND (CT Scan OR MRI OR Ultrasonography OR CBCT) AND (Scaphoid) from 2020-2024 and research articles only | 371 |

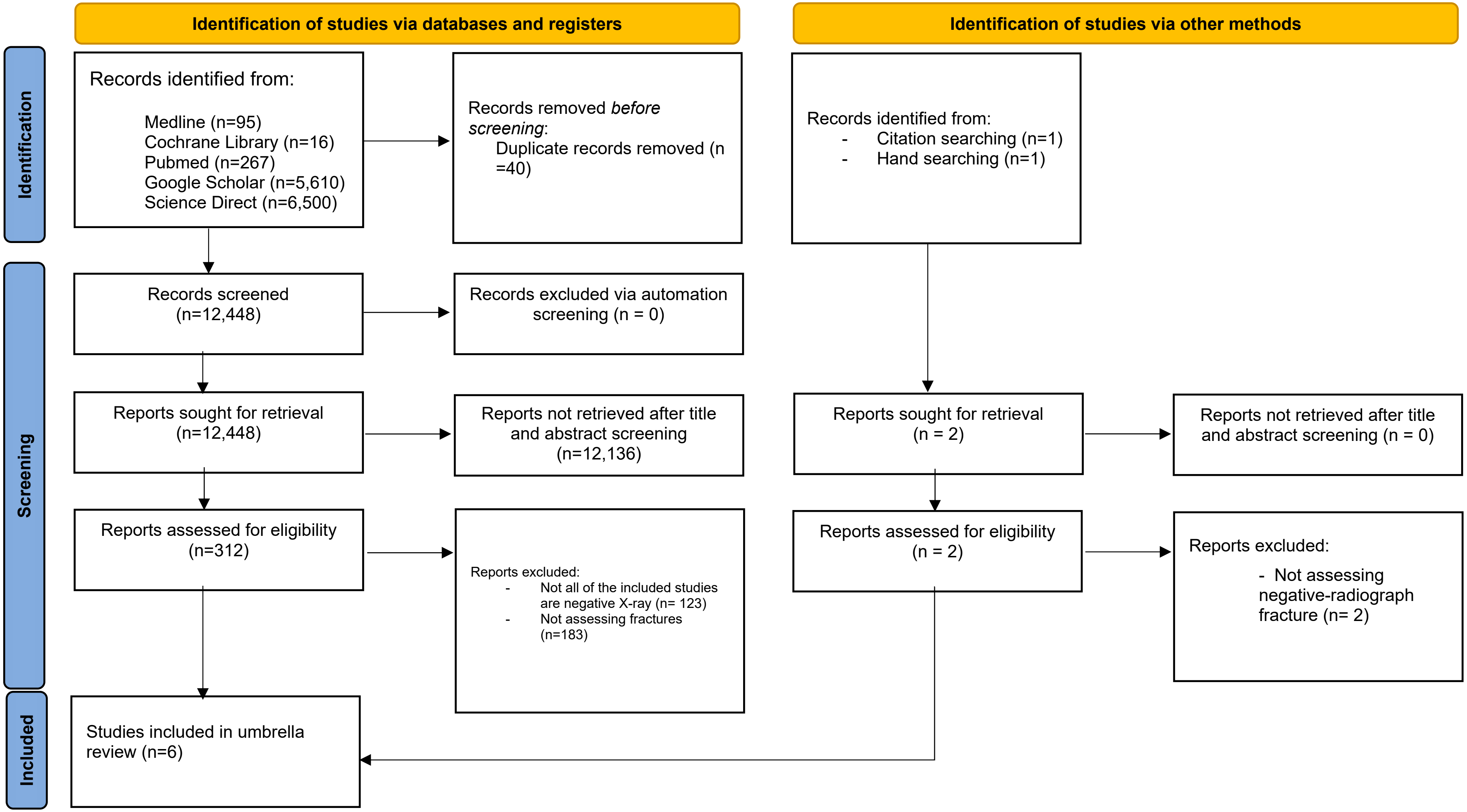


Supplementary Figure S1. PRISMA flowchart for selection of included studies in the umbrella review

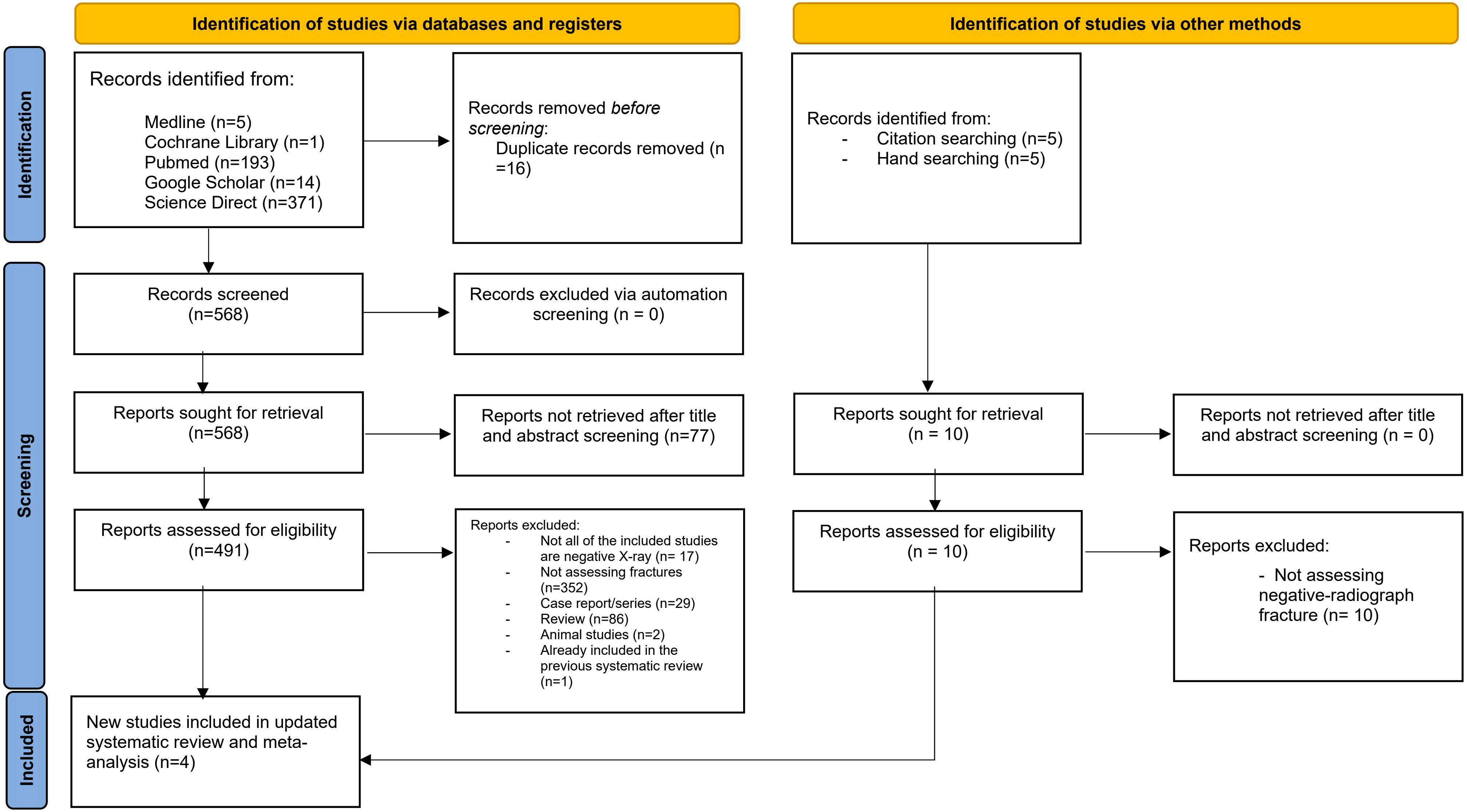


Supplementary Figure S2. PRISMA flowchart for selection of included studies in the systematic review of the occult fractures of the scaphoid

Supplementary Table S4. Notable exclusions for the umbrella review

| Author (Year) | Reason |
|---|---|
| Yang (2021)[(1)] | Out of the four articles included in this review, one study does not employ a reference test[(2)] (the study compared their finding to another study that used an MRI, and Yang (2021) mistook that as the study using MRI as a reference test) while another study did not perform the reference test on all patients[(3).] The other two studies in this review are used in the updated meta-analysis of diagnostic test accuracy |
| Najaf-Zadeh (2014)[(4)] | Out of nine articles included in this review, only four performed a reference test. |
| Joshi (2013)[(5)] | Not all of the primary studies include patients with initial negative X-rays |
| Sherbaf (2021)[(6)] | Not all of the primary studies include patients with initial negative X-rays |
| Gilbertson (2022)[(7)] | Not all of the primary studies include patients with initial negative X-rays |
| Adeyemo & Akadiri[(8)] | Not all of the primary studies include patients with initial negative X-rays |
| Fitzpatrick (2022)[(9)] | Not all of the primary studies include patients with initial negative X-rays |
| Duarte (2021)[(10)] | Compared low-dose CT scan to a standard-dose CT scan |
| Yin (2010)[(11)] | Not all of the primary studies include patients with initial negative X-rays |
| Offiah & Burke (2018)[(12)] | |
| Champagne (2019)[(13)] | |
| Wu (2021)[(14)] | |
| Yin (2012)[(15)] | |
| Alabousi (2019)[(16)] | |
| Gordon (2021)[(17)] | |
| Mennen (2023)[(18)] | |
| Gadicherla (2021)[(19)] | |
| Alexandridis (2022)[(20)] | |
| Malgo (2017)[(21)] | |
| Adeyemo & Akadiri (2011) | |

MRI; Magnetic resonance imaging; CT, Computed tomography

Supplementary Table S5. Notable exclusions for the scaphoid fracture

| Author (Year) | Reason |
|---|---|
| Jain (2018)[22] | Not all X-rays are negative |
| Nguyen (2021)[23] | No reference test was conducted |
| Daniels (2023)[24] | Not all X-rays are negative |
| Bulstra (2023)[25] | No reference test was conducted |
| Dean (2021)[26] | No reference test was conducted |
| Etli (2020)[27] | No reference test was conducted |
| Kim (2021)[28] | No negative x-rays |
| Kelson (2016)[29] | No reference test was conducted |
| Rolfe (1981)[30] | A 2x2 table information could not be extracted reliably |
| Wilson (1986)[31] | Not all patients underwent reference tests |

Supplementary Table S6. Descriptive characteristics of each study included in occult scaphoid fracture

| **Author (Year)** | **Sample size** | **Setting** | **Study type** | **Sampling** | **Inclusion criteria** | **Exclusion criteria** | **Index test** | **Index test machine** | **Reference test** | **Reference test machine** | **Age** | **Sex** | **Definition of negative fracture** | **Definition of fracture** |
|---|---|---|---|---|---|---|---|---|---|---|---|---|---|---|
| Yildirim (2013)[32] | 63 | ED | Prospective | Consecutive | Not mentioned | Not mentioned | Ultrasound | DC 3, Mindray Bio-medical Electronics Co., Shenzhen, China | MRI | Not mentioned | 39.6±18.3 | 52.4% female | Not mentioned | Cortical disruption of the scaphoid contour, producing a focal deformity, or hemarthrosis, seen as hypoechoic fluid in the radio scaphoid or scaphotrapezium trapezoid spaces |
| Platon (2011)[33] | 62 | ED | Prospective | Consecutive | Not mentioned | Not mentioned | Ultrasound | Prosound SSD-5000SV; ALOKA, Tokyo, Japan | CT | MX 8000 Philips Medical Systems, Best, The Netherlands | 41.2 years (18-89 years old) | 52.3% female | Not mentioned | Cortical disruption of the scaphoid contour produces a focal step of deformity, or hemarthrosis, seen as hypoechoic fluid in the radio scaphoid or scapho-trapezium-trapezoid spaces |
| Fusetti (2005)[34] | 24 | ED | Prospective | Consecutive | Not mentioned | Not mentioned | Ultrasound | ATL-Philips 5000 | CT | MX8000 16 Slices (Philips Medical System, Inc., Cleveland, Ohio) | Not mentioned | 45.8% female | Not mentioned | Scaphoid cortical interruption |

| | | | | | | | | | | | | | | |
|---|---|---|---|---|---|---|---|---|---|---|---|---|---|---|
| Senall (2004)[35] | 18 | ED | Prospective | Not mentioned | Not mentioned | Not mentioned | Ultrasound | ATL HDI 3000; Philips Medical Systems, Seattle, WA | Follow-up radiography | Not mentioned | 35 (10-77) | Not mentioned | Not mentioned | Healing callus or fracture site resorption |
| Hauger (2002)[36] | 54 | Not mentioned | Prospective | Consecutive | Not mentioned | Not mentioned | Ultrasound | HDI 3000 and 5000; Advanced Technology Laboratories, Bothell, WA | Follow-up radiography and clinical follow-up, CT (4 patients), MRI (1 patient) if suspicion of a fracture persisted | Not mentioned | 26 (10-75) | 35.2% female | Not mentioned | Visualization of follow-up radiographs of a fracture line through the bone, cortical disruption, or cortical avulsion |
| Herneth (2001)[37] | 15 | Not mentioned | Prospective | Consecutive | Not mentioned | Not mentioned | Ultrasound | HDI 3000; ATL, Bothell, Wash | MRI | 1.0-T unit (Gyroscan T10-NT; Philips, Best, the Netherlands) with a circular surface coil (C3; Philips, 14 external and 11 cm internal diameter) | 23.5 (15.8-55.2) | 53.3% female | Not mentioned | Not mentioned |
| Xie (2020)[38] | 20 | ED | Prospective | Not mentioned | Not mentioned | Not mentioned | DECT | Revolution CT (GE Medical Systems) | MRI | 3-T HDx 750 (GE Medical Systems) | 51 (20-76) | 20% female | Not mentioned | Not mentioned |
| Kitsis (1989)[39] | 22 | Casualty and other orthopaedic clinics to a special hand clinic. | Prospective | Not mentioned | Not mentioned | Not mentioned | MRI | Picker Vista 0.5 Tesla knee coil | Bone Scan | Injection of 550 Mbq of technetium 99m HDP. | 34 (15-72) | 59.1% female | Not mentioned | Not mentioned |
| Thorpe (1996)[40] | 59 | Not mentioned | Prospective | Consecutive | Not mentioned | Not mentioned | MRI | 1 Tesla Magnetom Impact (Siemens, Erlangen, Germany) | Bone Scan | Injection of 350-750 MBq of "Tcm methylene diphosphonate | 22 (12-71) | 64% female | Not mentioned | Not mentioned |
| Fowler (1998)[41] | 43 | Not mentioned | Prospective | Consecutive | Not mentioned | Not mentioned | MRI & Bone Scan | Siemens Impact 1.0 T machine & single-headed gamma camera 3 h after intravenous injection of 99Tc-mercapto diphosphonate | "GS diagnosis" When the MRI and BS-based diagnoses concurred this was taken to be the GS diagnosis. When there was a discrepancy between the MRI- and BS-based diagnoses, plain films (including follow-up films | Not mentioned | 32 (12-74) | 51.2% female | Not mentioned | MRI findings taken to represent a fracture were the presence of both marrow oedema (demonstrated best by the STIR images) and a trabecular fracture line (shown by the T1 and T2 images). The BS finding interpreted as a fracture was an intense focus of |

| | | | | | | | | | | | | | |
|---|---|---|---|---|---|---|---|---|---|---|---|---|---|
| | | | | | | | | | from the orthopaedic clinic when available) were used to resolve the dilemma and come to the GS diagnosis | | | | | activity emanating from a bone |
| Borel (2017)[42] | 49 | Not mentioned | Prospective | Not mentioned | Not mentioned | Not mentioned | CBCT | Planmeca ProMax 3D mid, Helsinki, Finland | MRI | 3-T unit (Magnetom Skyra, Siemens Healthcare, Erlangen, Germany), and a dedicated 16-channel wrist coil (Hand/wrist 16, Siemens Healthcare, Erlangen, Germany) | 34.5 (18-79) | Not mentioned | Not mentioned | Not mentioned |
| Edlund (2016)[43] | 71 | ED | Prospective | Consecutive | Not mentioned | Not mentioned | CBCT | Planmed Oy, Helsinki, Finland | MRI | GE Optima 450 W, 1.5 T (GE Healthcare, Waukesha, WI, USA) or on a GE Optima HDxt Edition 23, 1.5 T (GE Healthcare, Waukesha, WI, USA) scanner | Not mentioned | 40% female | Not mentioned | Presence of a low signal line on T1 images in combination with a corresponding area of high signal on the STIR sequence |
| De Zwart (2012)[44] | 159 | ED | Prospective | Consecutive | Suspected scaphoid fracture (tender anatomic snuffbox and pain in the snuffbox when applying axial pressure on the first or second digit), a recent trauma (within 48 hours), and no evidence of a fracture on scaphoid radiographs. | Polytrauma patients, patients younger than 18 years and those with contraindications for bone scintigraphy or CT were excluded | CT & Bone Scan | CT (General Electric Lightspeed Qx/I CT Scanner, Pewaukee, WI) and Bone Scan (SKYLight gamma camera (Philips, Eindhoven, The Netherlands). | Pre-determined reference standard (look at the paper) | Not mentioned | 41 (17-88) | 50.3% female | Not mentioned | Not mentioned |

| | | | | | | | | | | | | | | |
|---|---|---|---|---|---|---|---|---|---|---|---|---|---|---|
| Beeres (2008)[45] | 100 | ED | Prospective | Consecutive | Had a suspected scaphoid fracture (tender in the anatomical snuffbox, and pain in the snuffbox when applying axial pressure to the thumb or index fingers, a recent injury (within 48 hours), and no evidence of a fracture on plain radiographs | Polytrauma patients, patients under the age of 18 years, and those in whom MRI was contraindicated were excluded | MRI & Bone Scan | MRI (1.5 Tesla MR scan (Siemens, Erlangen, Germany) and Bone Scan (Sky-Light gamma camera (Philips, Eindhoven, The Netherlands)) | Pre-determined reference standard (look at the paper) | Not mentioned | 42 (18-84) | 50% female | Not mentioned | Not mentioned |
| Ilica (2011)[46] | 54 | Not mentioned | Prospective | Not mentioned | Not mentioned | Admitted more than 72 hours after the trauma or <18 years old | CT | 64-detector multislice system (Brillance 64; Philips, Best, The Netherlands). | MRI | Signa 1.5-T MR system (GE Medical Systems, Milwaukee, WI, USA) | 22 (20-40) | 0% female | Without a sharp radiolucent line in the trabecular pattern, a distinct break of the cortex, or a sharp step-off in the cortex | MRI (evidence of a cortical fracture line, a trabecular fracture line, or a combination of these abnormalities) |
| Mallee (2011)[47] | 40 | Not mentioned | Prospective | Consecutive | Present within twenty-four hours after injury, have tenderness in the anatomic snuffbox | <18 years old, any concurrent distal ulnar, radial, or carpal fracture; previous scaphoid fracture; rheumatoid arthritis; and cognitive dysfunction that would limit the physical examination | CT and MRI | MRI (1.0-Tesla open MRI system (Panorama LOT; Philips Medical Systems, Eindhoven, The Netherlands) CT (Brilliance CT scanner (64 slices; Philips Medical Systems) | Scaphoid-specific radiographs six weeks after the initial scan | Not mentioned | Not mentioned | 37.5% female | Not mentioned | An abnormal lucent line within the scaphoid (X-ray) presence of a sharp lucent line within the trabecular bone pattern, a break in the continuity of the cortex, a sharp step in the cortex, or a dislocation of bone fragment (CT) presence of a cortical fracture line, a trabecular fracture line, or a combination of both (MRI) |
| Memarsadeghi (2006)[48] | 29 | Not mentioned | Prospective | Not mentioned | Not mentioned | Not mentioned | CT and MRI | Xray (Horizontal Diagnostic Super 80 CP; Philips Medical Systems, | Two radiologists in consensus | Not mentioned | 34 (+/-13) | 41.3% female | Not mentioned | MR (presence of a cortical fracture line, a trabecular |

| | | | | | | | | | | | | | | |
|---|---|---|---|---|---|---|---|---|---|---|---|---|---|---|
| | | | | | | | | Eindhoven, the Netherlands) MRI (1.0-T unit (Gyroscan T10-NT; Philips Medical Systems) MDCT (Somatom Volume Zoom; Siemens, Forchheim, Germany) | | | | | | fracture line, or a combination of both. Such a fracture line had to be evidenced by linear disruption of the normal trabecular pattern and had to be hyperintense on STIR and T2-weighted images and hypointense or hyperintense on T1-weighted images). CT (presence of a sharp lucent line within the trabecular bone pattern, a break in the continuity of the cortex, a sharp step in the cortex, or a dislocation of bone fragments) |
| Breitenseher (1997)[49] | 42 | Clinic for trauma surgery | Prospective | Consecutive | Not mentioned | Not mentioned | MRI | 1.0-1unit(Gyroscan T10-NT;Philips, Best,The Netherland) | Follow-up x-ray after 6 weeks | Not mentioned | 30.5 (+/-13.8) | 45.2% female | Not mentioned | Cortical fracture line, trabecular fracture line, a bone marrow abnormality that involved a diffuse area of a wrist bone, or the combination of 2 or 3 of these signs (MRI) X-Ray --> Sclerotic line and/or resorption around the fracture line |
| Tiel-van Buul (1996)[50] | 16 | Department of traumatology | Prospective | Not mentioned | Not mentioned | Not mentioned | MRI | Magnetom 63SP/4000, 1.5 T | Bone scan | Not mentioned | 36 (24-60) | 31.25% female | Not mentioned | Not mentioned |
| Nielsen (1983)[51] | 100 | Not mentioned | Prospective | Not mentioned | Not mentioned | Not mentioned | Bone scan | Nuclear Chicago Pho/Gamma 3 scanner | Clinical and radiographical examination 2 months after | Not mentioned | 33 (10-80) | 39% female | Not mentioned | Focal uptake radially or centrally in the wrist |
| O'Carroll (1982)[52] | 30 | Not mentioned | Prospective | Not mentioned | Not mentioned | Not mentioned | Bone scan | Not mentioned | X-ray | Not mentioned | 32 (11-72) | 30% female | Not mentioned | Not mentioned |
| Stordahl (1984)[53] | 30 | Not mentioned | Prospective | Not mentioned | Not mentioned | Not mentioned | Bone scan | Pho/Gamma 4 Camera with divergent low energy collimator, or pinhole collimator | X-ray 2 weeks and 6 weeks | Not mentioned | 31 (10-69) | 40% female | Symmetrical activity in both wrists, the same | Increased radioactivity, focal or diffuse |

| | | | | | | | | | | | | | | |
|---|---|---|---|---|---|---|---|---|---|---|---|---|---|---|
| | | | | | | | | | | | | | activity present over the distal ends of the Not mentioned radius and ulna, and even activity over the area of the palm | |
| Tiel-van Buul (1993)[54] | 125 | Not mentioned | Prospective | Consecutive | Not mentioned | Not mentioned | Bone scan | Not mentioned | Follow-up radiography day 10-14 and 6 weeks after | Not mentioned | 38.6 (12-84) | 51.25% female (out of 160)* | Not mentioned | Focally increased activity (a hotspot) in the scaphoid region in both the dynamic and the static images. |

ED; Emergency department, MRI, Magnetic resonance imaging, CT, Computed tomography

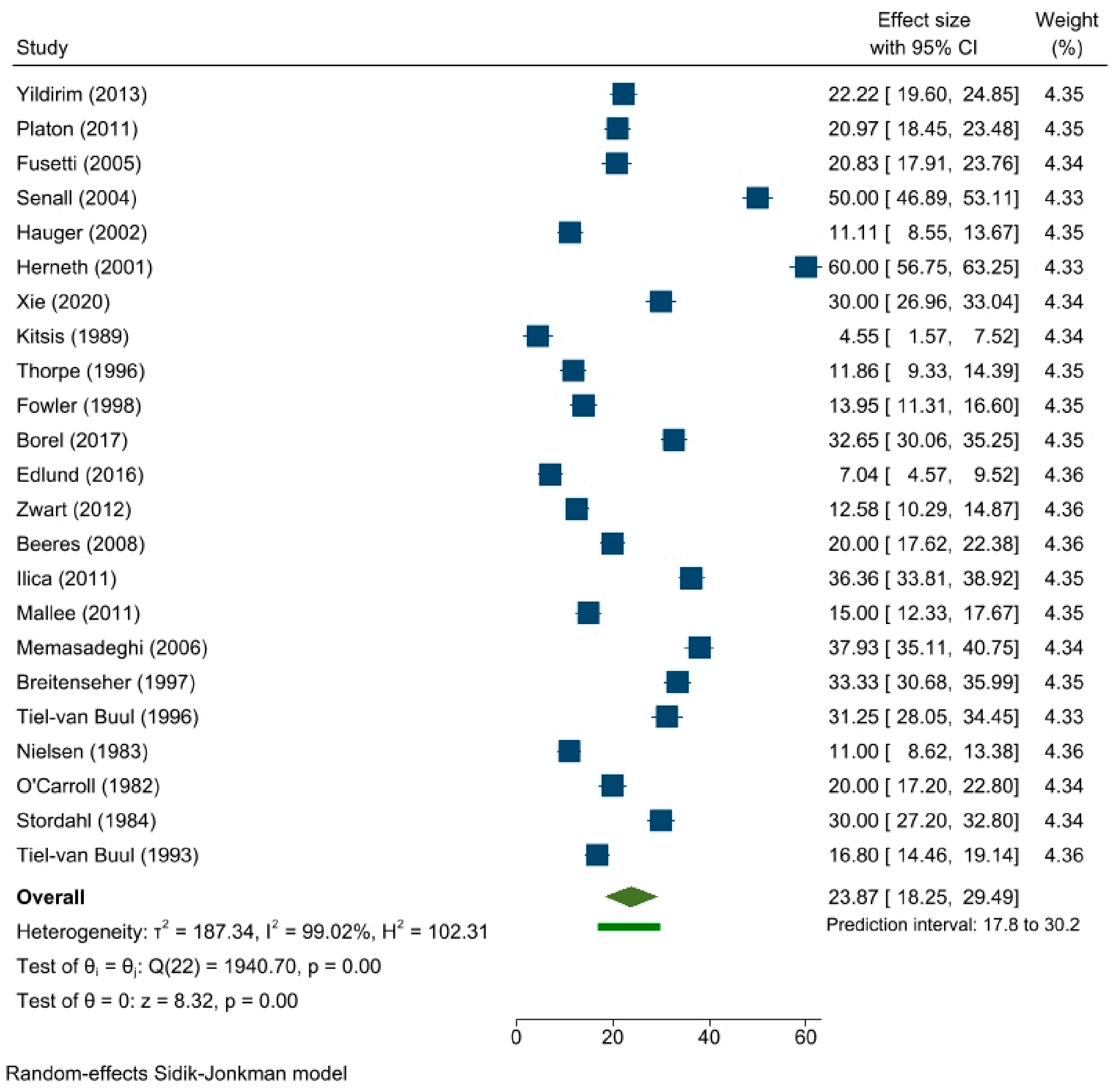


Supplementary Figure S3. Meta-Analysis of Prevalence of Occult Scaphoid Fracture

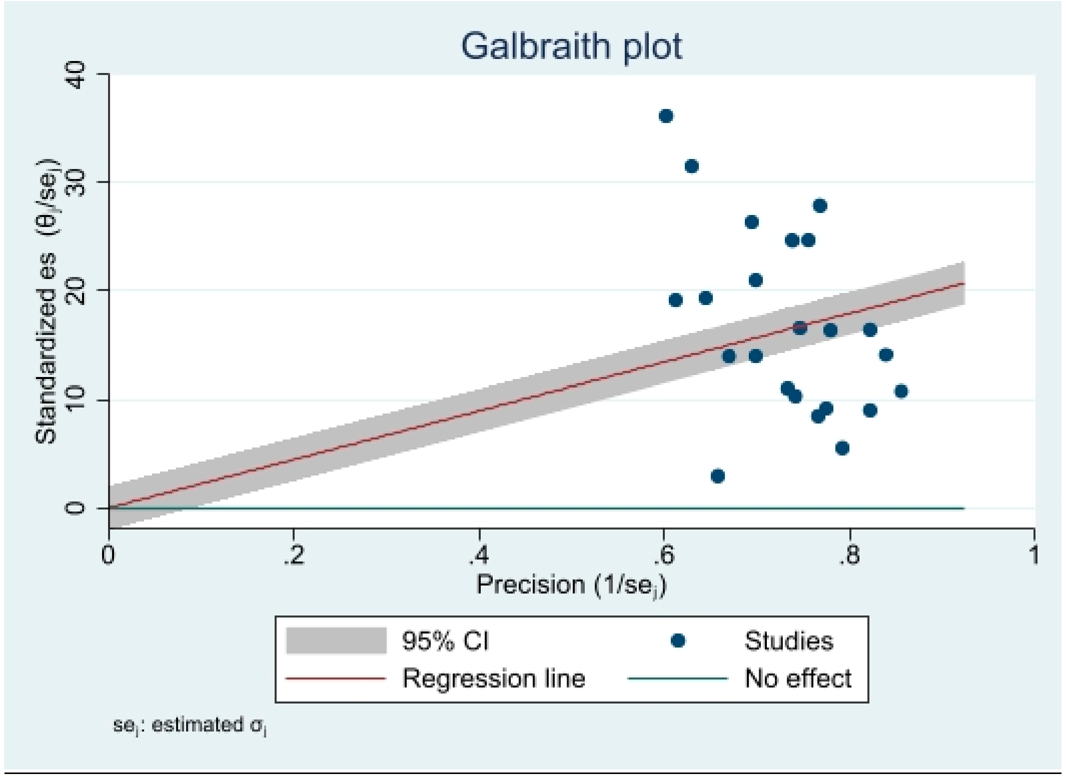


Supplementary Figure S4. Galbraih plot of occult scaphoid fracture

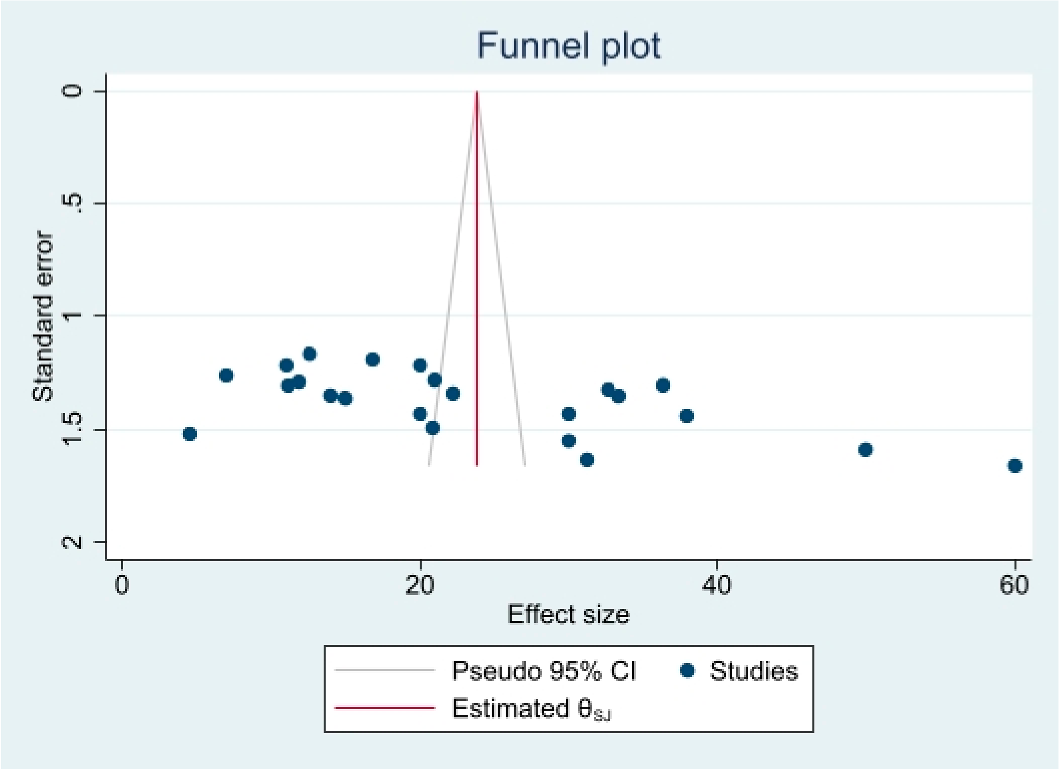


Supplementary Figure S5. Funnel plot of studies included in the prevalence of occult scaphoid fracture

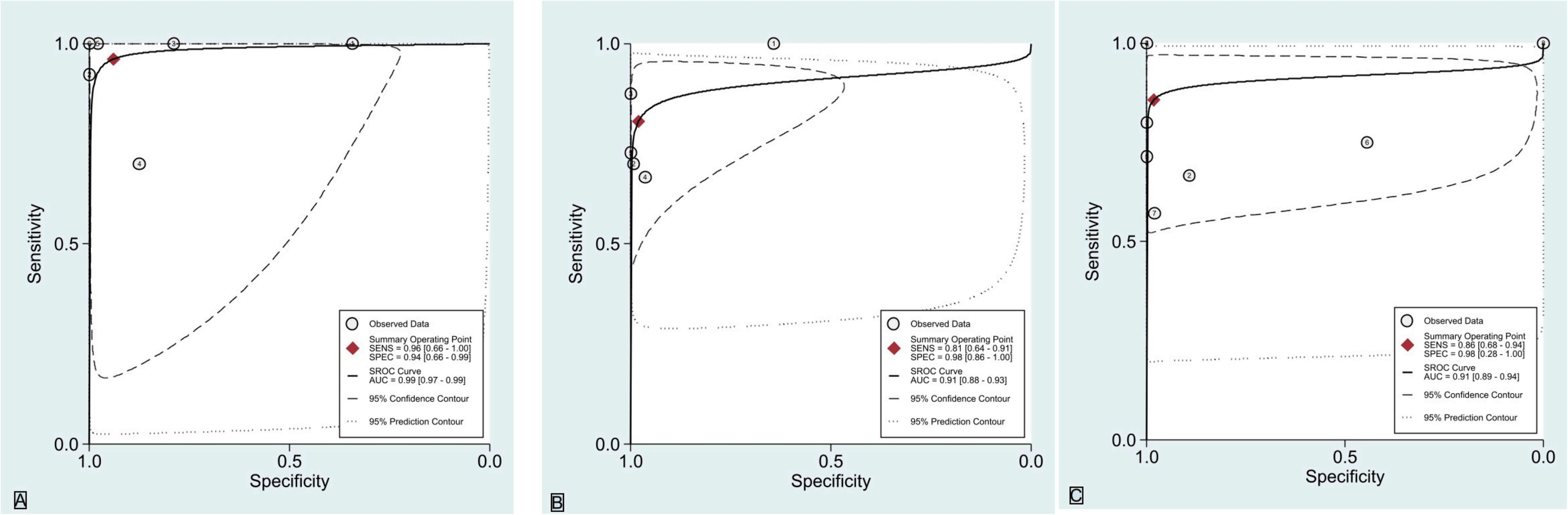


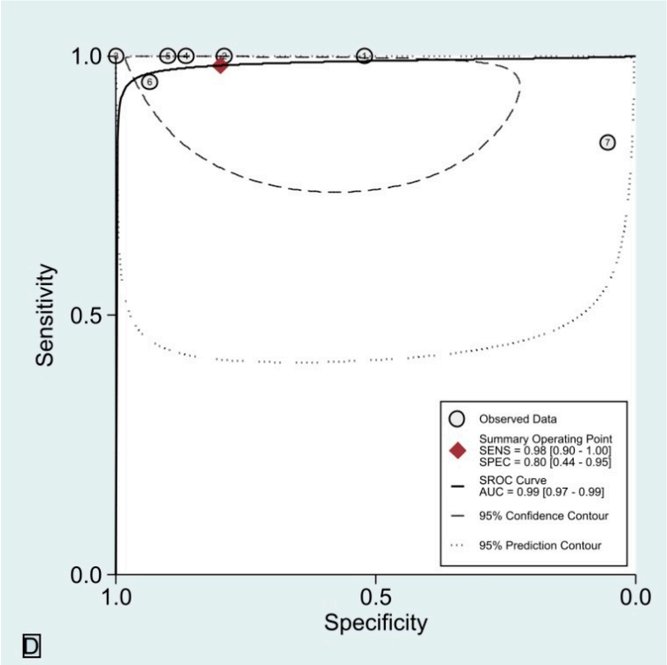


Supplementary Figure S6. The hierarchical summary receiver operating characteristic (HSROC) of ultrasound (A), CT (B), MRI (C), and bone scan (D) in detecting occult scaphoid fracture

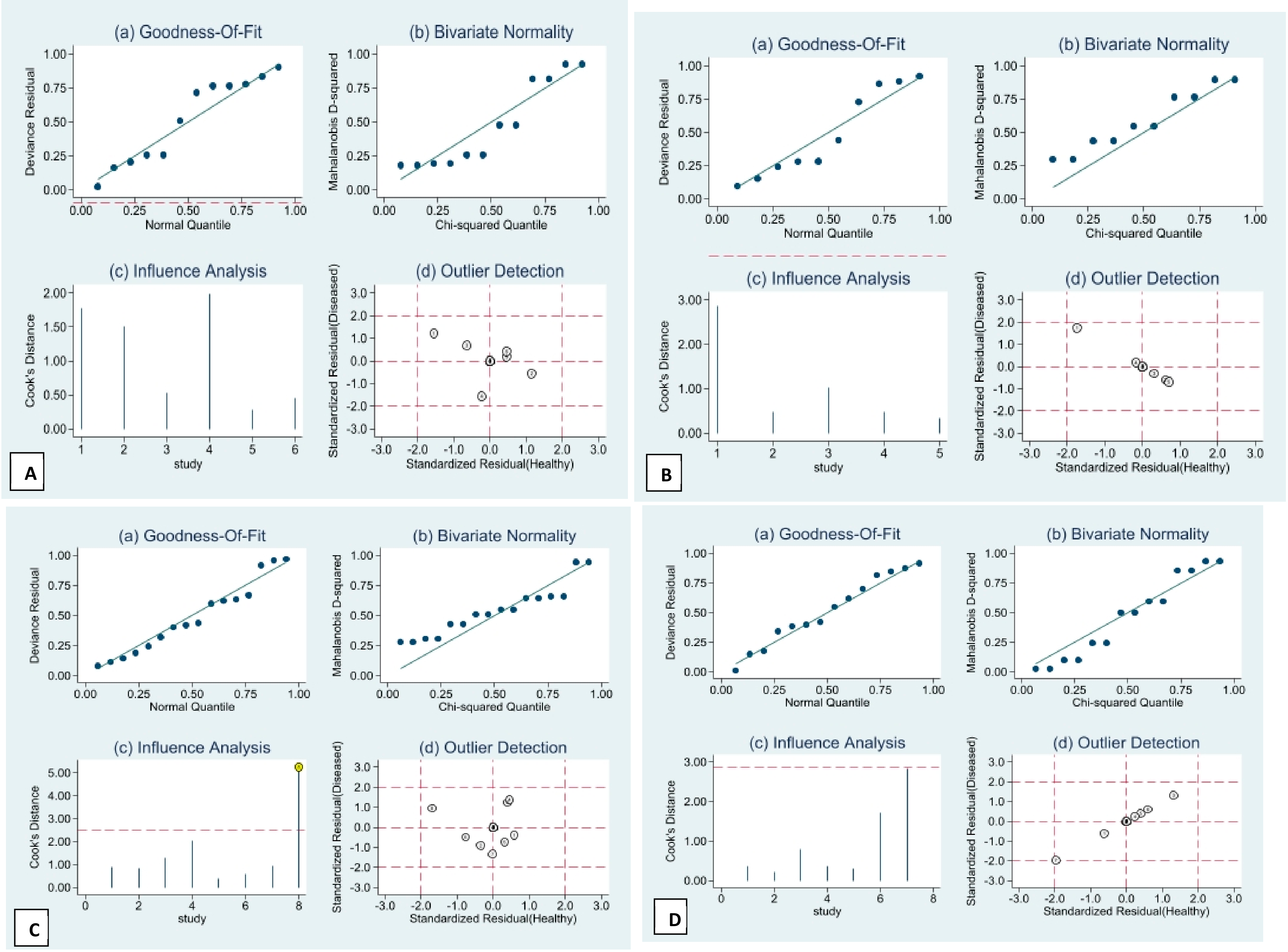


Supplementary Figure S7. Model diagnostics of each study for ultrasound (A), CT (B), MRI (C), and bone scan (D) in detecting occult scaphoid fracture

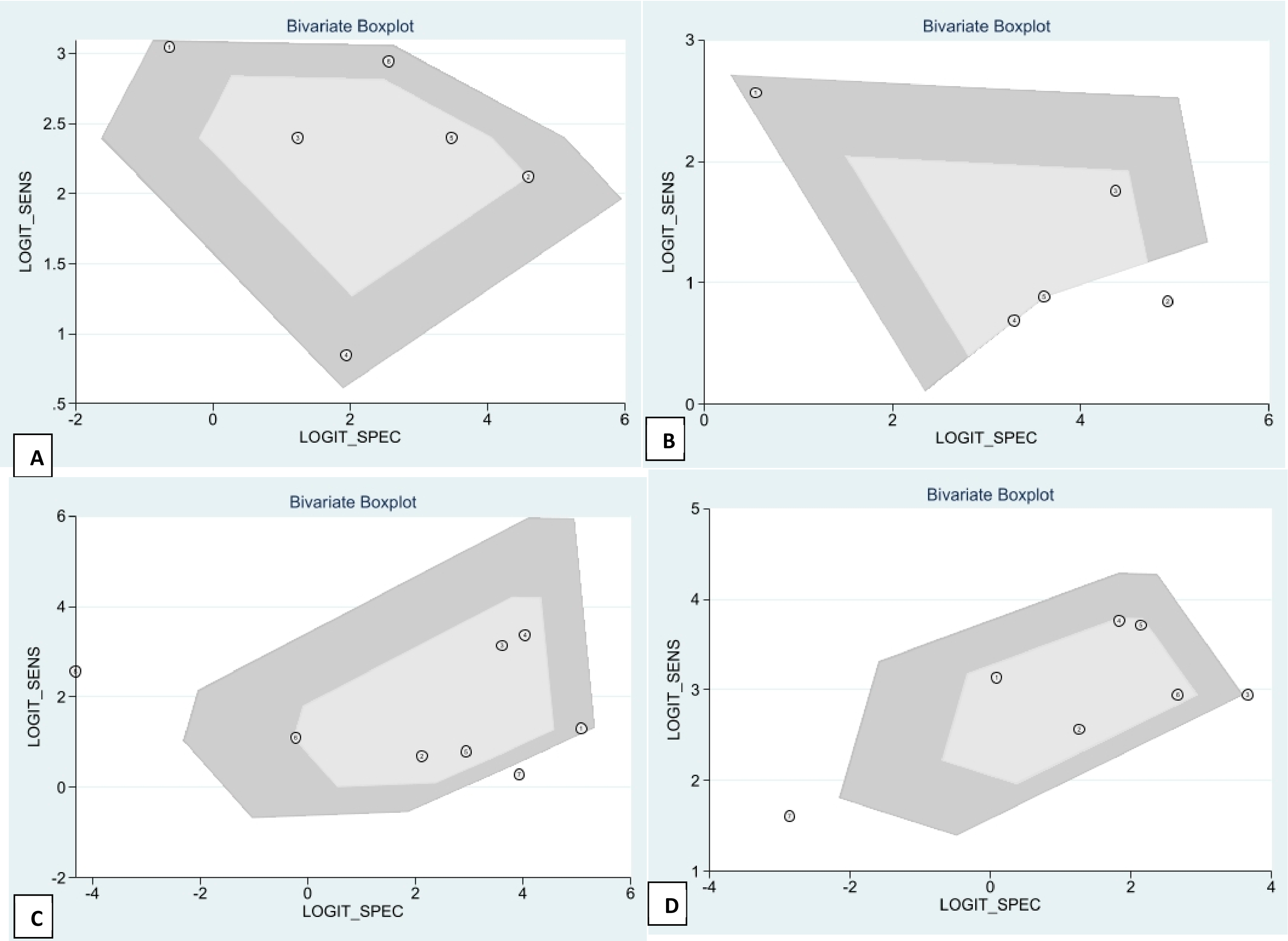


Supplementary Figure S8. Bivariate boxplot of each study for ultrasound (A), CT (B), MRI (C), and bone scan (D) in detecting occult scaphoid fracture

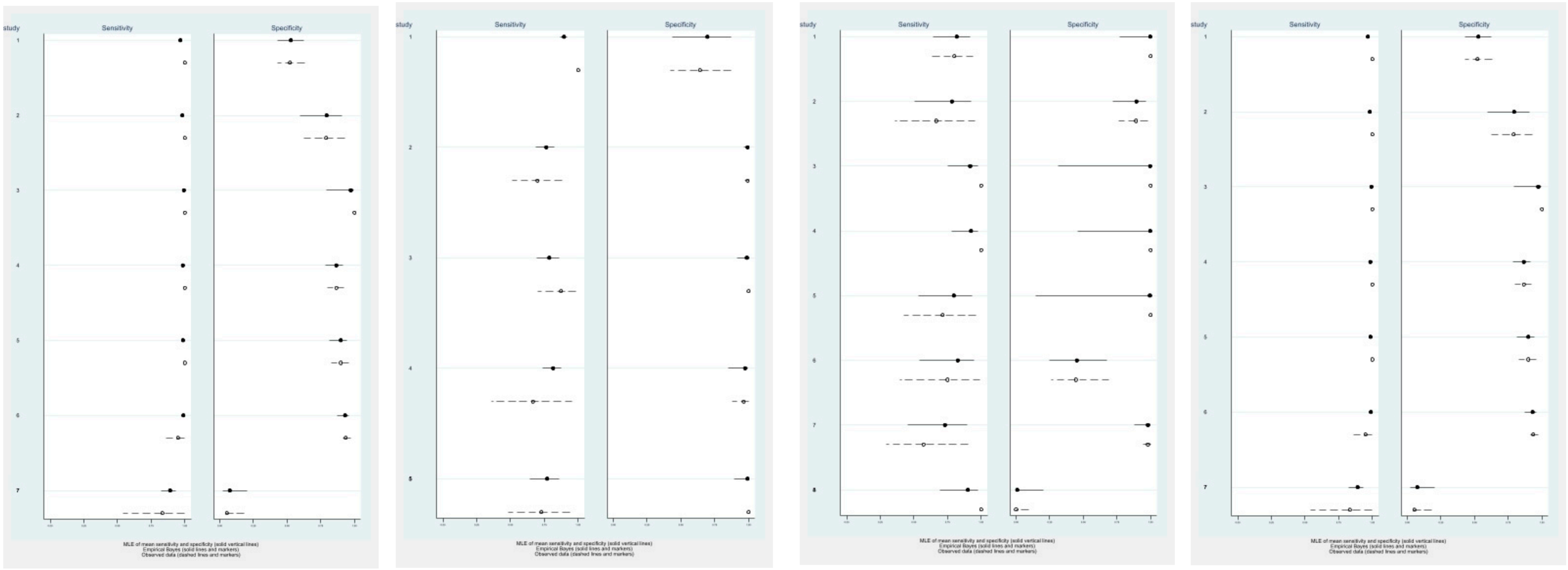


Supplementary Figure S9. Empirical Bayes prediction of sensitivity and specificity of each study for ultrasound (A), CT (B), MRI (C), and bone scan (D) in detecting occult scaphoid fracture

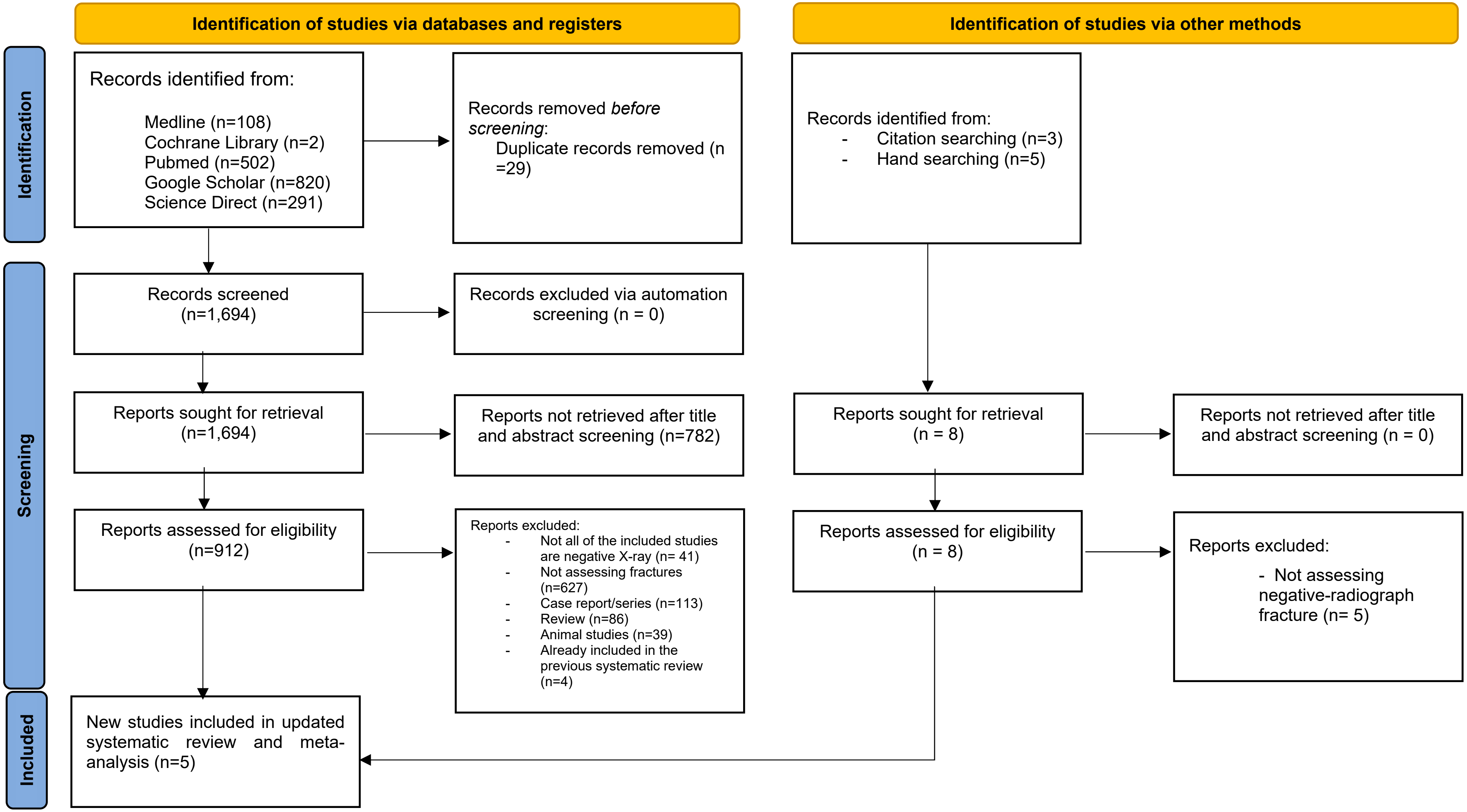


Supplementary Figure S10. PRISMA flowchart for selection of included studies in the systematic review of the occult fractures of the hip

Supplementary Table S7. Descriptive characteristics of each study included in occult scaphoid fracture

| Author (Year) | Sample size | Setting | Study type | Sampling | Inclusion criteria | Exclusion criteria | Index test | Index test machine | Reference test | Reference test machine | Age | Sex | Definition of negative fracture | Definition of fracture |
|---|---|---|---|---|---|---|---|---|---|---|---|---|---|---|
| Deutsch (1989)[55] | 23 | ED | Retrospective | Not Mentioned | Clinical suspicion of fracture, normal radiographs | Not mentioned | MRI | 1.5-T superconducting magnet (Signa; General Electric Medical Systems, Milwaukee) | Clinical follow-up in three months | N/A | 66-84 | 65.5% Female | Not mentioned | Linear or oblique line of decreased signal intensity on a T1-Weighted image that was surrounded by a broader, poorly defined, less dark area. |
| Rizzo (1993)[56] | 62 | Not mentioned | Prospective | Not Mentioned | A fracture of the hip was clinically suspected, but in whom the radiographic findings were negative | Not mentioned | Bone scan | Not mentioned | MRI | N/A | Not mentioned | Not mentioned | Not mentioned | Not mentioned |
| Quinn & McCarthy (1993)[57] | 20 | Good Samaritan Hospital and Medical Center | Prospective | Consecutive | Suspected hip fracture with intermediate radiographs | Not mentioned | MRI | 1.5-T superconducting MR unit (Signa; GE Medical System) | Clinical Follow-Up | N/A | 78 | 95% Female | Not mentioned | Not mentioned |
| Evans (1994)[58] | 37 | Not mentioned | Retrospective | Not mentioned | Elderly, hip pain after fall, normal radiographs | Not mentioned | MRI & Bone Scan | Not mentioned | Follow up | N/A | Not mentioned (elderly) | Not mentioned | Not mentioned | Not mentioned |
| Haramati (1994)[59] | 15 | Not mentioned | Prospective | Not mentioned | Osteopenia, Normal radiographs, suspected hip fracture. | Radiographs demonstrate a fracture | MRI | 1.5-T MR unit using the body coil (Philips Medical Systems, Eindhoven, Netherlands). | Clinical and surgical follow-up | N/A | 70.9 | 73% Female | Not mentioned | Not mentioned |
| Bogost (1995)[60] | 70 | Not mentioned | Retrospective | Consecutive | History of trauma or rule out fracture | Hip pain of unknown origin | MRI | 1.5-T MR unit (Signa; GE Medical Systems, Milwaukee, Wis). | Clinical follow-Up | N/A | 76 | 70% Female | Not mentioned | Not mentioned |
| Stiris and Lilleas (1997)[61] | 27 | Not mentioned | Prospective | Not mentioned | Not mentioned | Not mentioned | MRI | superconducting magnet operating at 1.0 T (Magnetom Expert, Siemens | Clinical follow-up | N/A | 76.9 | 81% Female | Not mentioned | Fracture line involving the cortical rim |
| Rubin (1998)[62] | 40 | ED | Retrospective | Not mentioned | Clinically suspected hip fracture, negative or equivocal plain films, and either a subsequent bone scan or MRI examination were | Not mentioned | MRI or bone scan | 1.5-T clinical unit (GE Signa, Milwaukee, Wis.) | Follow up in three months | N/A | 28-99 | 72.5% Female | Not mentioned | Not Mentioned |

| | | | | | | | | | | | | | | |
|---|---|---|---|---|---|---|---|---|---|---|---|---|---|---|
| | | | | | retrospectively reviewed | | | | | | | | | |
| Pandey (1998)[63] | 33 | ED | Not mentioned | Not mentioned | Post-traumatic hip pain, negative radiographs | Not mentioned | MRI | GE 1.5T Sigma MRI scanner | Clinical follow-up | N/A | 72 | Not mentioned | Not mentioned | Not mentioned |
| Lim (2002)[64] | 57 | ED | Not mentioned | Not mentioned | Suspected fracture, negative or equivocal x-ray | Fracture visible on x-ray, metallic implant, prosthesis | MRI | Magnetom Vision (Siemens Medical Systems, Germany) 1.5 Tesla scanner | Clinical follow-up | N/A | 71.8 | 25% Female | Not mentioned | Not mentioned |
| Oka & Monu (2004)[65] | 73 | Not mentioned | Retrospective | Not mentioned | Negative or equivocal radiographs | Inadequate radiographs | MRI | 1.5-T unit (Signa, General Electric Medical Systems, Milwaukee, WI) | Clinical follow-up | N/A | 67 | 61% Female | Not mentioned | The linear low-signal focus was surrounded by an intermediate signal area on T1-weighted images and the linear low-signal focus was surrounded by a high signal on T2-weighted images |
| Galloway (2004)[66] | 70 | ED | Retrospective | Consecutive | Patients over 50, inability to weight bear following a fall, initial radiograph normal | Not mentioned | MRI | a Siemens Vision 1.5T (Siemens, Erlangen, Germany) | Clinical follow-up | N/A | 81.1 | 65.7% Female | Not mentioned | Not mentioned |
| Lee (2004)[67] | 28 | ED | Retrospective | Not mentioned | A clinically suspected traumatic femoral neck fracture, no fracture visible radiograph, patient undergoing MRI exam. | Not mentioned | MRI | 1.5-T Gyroscan machine (Philips, Best, Netherlands); T1-weighted (time-to-repetition [TR], 590; time-to-echo [TE], 20), and T2-weighted fat-suppressed short-tau inversion recovery (STIR) sequence (TR, 5170; TE, 56) in the oblique coronal plane were obtained using a standard surface coil | Clinical and surgical follow-up | Not mentioned | 69-93 | 89% Female | Not mentioned | Hypointense line transversing the medullary canal on T1-weighted images. |
| Alam (2005)[68] | 18 | Hospital | Retrospective | Not mentioned | Suspected hip fracture, normal plain radiographs, undergone MRI. | Not mentioned | MRI | 1.5T magnet (GE Medical Systems, Milwaukee, Wisconsin) | Clinical follow-up | Not mentioned | Not mentioned | Not mentioned | Not mentioned | Not mentioned |
| Frihagen (2005)[69] | 100 | Not mentioned | Prospective | Consecutive | Not mentioned | Not mentioned | MRI | Not mentioned | Clinical follow-up | Not mentioned | Not mentioned | Not mentioned | Not mentioned | Not mentioned |

| | | | | | | | | | | | | | | |
|---|---|---|---|---|---|---|---|---|---|---|---|---|---|---|
| Verbeeten (2005)[70] | 33 | ED | Not mentioned | Not mentioned | Clinically suspected hip fracture, negative or equivocal radiographs, subsequent MRI. | Not mentioned | MRI | Panorama 0.23 T, Panorama 0.6T, Infinion | Surgery for the insertion of a dynamic hip screw | (Medical Record) | 79 | 84.8% Female | Not mentioned | In X-ray images, a fracture was indicated as present if a sclerotic line or any cortical/trabecular discontinuities were observed. In MR images, a fracture was indicated as present if there was a linear low signal area from cortex to cortex on T1W sequences |
| Lubovsky (2005)[71] | 13 | University Hospital | Not mentioned | Not mentioned | Patients with suspected hip fractures and no evidence of fractures on radiographs. | Not mentioned | CT & MRI | MX8000 multislice helical scanner (manufactured by Phillips Medical System, Best, Netherlands), axial 3.2 mm with a pitch of 0.5 | MRI | T1 weighted spine echo + STIR (Short Tau Inversion Recovery) axial and coronal scan, a Torso Array Coil with a slice thickness of 3 mm with a gap of 1 mm and 1.5 Tessla Sigma-LX (manufactured by GE Medical Systems, Milwaukee, MN, USA). | 73 | 84.6% Female | Not mentioned | Not mentioned |
| Dominguez (2005)[72] | 62 | ED | Not mentioned | Not mentioned | Hip pain + ED Visit + Negative radiographs | Not mentioned | MRI | Not mentioned | Clinical follow-up | Not mentioned | 66.7 (± 20.6) | 62.3% Female | Not mentioned | 1) Fracture evident on initial plain radiograph, 2) fracture evident on MRI of the hip performed at the time of ED care, or 3) subsequent diagnosis of hip and/or pelvic fracture as determined through follow-up |
| Chana (2006)[73] | 35 | ED | Prospective | Not mentioned | Hip pain + suspicion of fracture + normal radiographs | Not mentioned | MRI | Not mentioned | Surgical follow-up | Not mentioned | 81 | 66.6% Female | Not mentioned | Not mentioned |
| Hossain (2007)[74] | 57 | Not mentioned | Retrospective | Not mentioned | Suspected hip fracture, normal x-ray, undergo MRI. | Suspected stress fracture, metastatic | MRI | Phillips 1.5 T Intera Upgrade Magnet, with | Surgical follow-up | Not mentioned | 80 | 83% Female | Not mentioned | Not mentioned |

| | | | | | | | | | | | | | | |
|---|---|---|---|---|---|---|---|---|---|---|---|---|---|---|
| | | | | | | fracture, inadequate or untraceable clinical notes, osteoarthritis of the hip joint, fibromyalgia, and multiple myeloma. | | Coronal T1W/ TSE, T2W/TSE and STIR sequences performed as routine | | | | | | |
| Sankey (2009)[75] | 98 | Not mentioned | Prospective | Consecutive | A history of falls, unable to bear weight on the affected side, negative plain radiographs, and high clinical suspicion of fracture. | Unable to take an MRI | MRI | Siemens 1T system (Magnetom Impact, Siemens AG, Erlangen, Germany) between 1997 and 2002, and a 1.5T Siemens Symphony MR Scanner (Siemens Medical Solutions, Erlangen, Germany) | Clinical follow-up | Not mentioned | 77 (42-100) | 75.5% Female | Not mentioned | Not mentioned |
| Safran (2009)[76] | 30 | University Hospital | Prospective | Not mentioned | (1) Difficulty or inability to bear weight after a fall, (2) tenderness around the hip with a painful hip motion, and (3) negative pelvic and hip radiographic findings (both anteroposterior and lateral views) for the presence of hip and pelvic fractures. | (1) Prior ipsilateral hip fractures or surgery and (2) contraindications to MRI | US | HDI 5000 ultrasound device (Philips Healthcare, Bothell, WA) with SonoCT and harmonic imaging capabilities | MRI | 1.5-T Signa scanner (GE Healthcare, Milwaukee, WI) or a 1.5-T Avanto scanner (Siemens AG, Erlangen, Germany). | 73 | 80% Female | Not mentioned | Not mentioned |
| Szewczyk (2012)[77] | 102 | Not mentioned | Retrospective | Not Mentioned | no confounding co-morbidity, a genuine history of low-energy trauma, contemporaneous MRI and radiographic examinations, and an MRI that was performed due to clinical suspicion of an undiagnosed fracture | No expressed clinical suspicion of occult fracture; history suggesting high-energy trauma; skeletal co-morbidity hindering acute fracture identification; interval more than 2 weeks between radiographs and MRI. | MRI | 1.5-T MRI scanners (Siemens, Erlangen, Germany) | Clinical follow-up | Not mentioned | 82 | Not mentioned | Not mentioned | Not mentioned |

| | | | | | | | | | | | | | | |
|---|---|---|---|---|---|---|---|---|---|---|---|---|---|---|
| Iwata (2012)[78] | 35 | Not mentioned | Retrospective | Consecutive | Normal radiographs, MRI within 48 hours | Not mentioned | MRI | Siemens 1T system (Harmony MR Scanner; Siemens, Munich, Germany) between January 2003 and February 2004, a Siemens 1.0T system (Magnetom Impact; Siemens) between March 2004 and June 2010, and a Toshiba 1.5T system (Excelart Vantage; Toshiba, Tochigi, Japan) | Clinical follow-up | Not mentioned | 79.5 | 84% Female | Not mentioned | Not mentioned |
| Dunker (2012)[79] | 193 | ED | Retrospective | Not Mentioned | Suspected hip fracture at pelvis and hip radiography after low-energy trauma or no detected hip fracture but remaining clinical concern, patient age above 60, CT performed within 24 hours of radiography | Not mentioned | CT | 16 (Somatom Sensation, Siemens, Erlangen, Germany) or 40 detector rows (Philips Brilliance 40, Eindhoven, The Netherlands) | A combination of clinical information and MRI | Not mentioned | 83 | 62.7% female | Not mentioned | Not mentioned |
| Ohishi (2012)[80] | 113 | ED | Retrospective | Not Mentioned | Suspect fracture, normal radiographs, undergone MRI Scan | MRI more than 48 hours, MRI not clear for interpretation. | MRI | A 0.5-T unit (Flexart, TOSHIBA, Japan) in 39 cases between 2004 and 2006 and a 1.5-T unit (Excelart Vantage, TOSHIBA, Japan) in 74 cases thereafter. | Clinical follow-up | Not mentioned | 82,1 | 80.5% Female | Not mentioned | Linear, focal or broad-based the low-intensity area on T1-weighted MR images and a lineal low-intensity line surrounded by a high-intensity area at the corresponding focus on T2-weighted images or STIR sequence |
| Geijer (2012)[81] | 231 | Two hospitals | Retrospective | Not mentioned | Patients age 60 years or older, CT examination within | Not mentioned | CT | 16 (Somatom Sensation, Siemens, Erlangen, | Clinical follow-up | Not mentioned | 83 | 64.5% female | Not mentioned | Not mentioned |

| | | | | | | | | | | | | | | |
|---|---|---|---|---|---|---|---|---|---|---|---|---|---|---|
| | | | | | 10 days (mean 0.9 days) of trauma | | | Germany) and 40 detector rows (Brilliance 40, Philips, Eindhoven, The Netherlands) | | | | | | |
| Gill (2013)[82] | 92 | One hospital | Retrospective | Not mentioned | Patients presenting with clinical suspicion of a hip fracture (persistent hip pain after trauma, inability to bear weight, and pain on attempted straight leg raising, passive rotation, or axial loading tests), Patients initially underwent both the anteroposterior pelvis and lateral hip radiographs. If these were negative or inconclusive, further imaging was then arranged | Not mentioned | CT or MRI | CT: Siemens scanners with a ×4 quad slice and ×1 62 slice or MRI: Philips 3T or a Philips 1.5T. | Surgical and/or clinical follow-up | Not mentioned | 82 | 64.1% female | Not mentioned | Not mentioned |
| Heikal (2014)[83] | 65 | ED | Retrospective | Non-Consecutive | A low-impact trauma in whom fractured NOF was suspected despite a normal x-ray. | Not mentioned | CT | Not mentioned | Clinical follow-up | Not mentioned | 81.2 | 64.6% female | Not mentioned | Not mentioned |
| Haubro (2015)[84] | 67 | ED | Prospective | Consecutive | Hip pain after low-energy trauma after falling against the hip region but no fracture on standard X-rays were included. Underwent CT and/or MRI. | Incomplete medical records, registered two times, lack of cooperation to scanning. | CT Scan | GE 4 slice VCT scanner, GE 1 slice CT scanner, GE XTlight Speed VCT 64 slice. Scan protocol: Helical, full. 0.625 mm with a pitch of 0.984, image interval 0.625 mm. 120 kV, 700 mA. | MRI | Phillips 3T Acieva, Phillips 1T Panorama (open), and Phillips 1.5T Acieva | 80.5 | 59.7% female | Not mentioned | X-ray: A continuous fracture line from cortex to cortex on one projection<br>CT: A continuous fracture line from cortex to cortex<br>MRI: A low signal line in the T1W sequence and a corresponding high signal in the STIR sequence from cortex to cortex |
| Deleanu (2015)[85] | 35 | ED | Retrospective | Not Mentioned | Patients with fractures with negative Radiographs and CT/MRI on initial imaging. | Not mentioned | CT Scan | Not mentioned | Clinical information, CT Scan, and MRI | Not mentioned | Not mentioned | Not mentioned | Not mentioned | Not mentioned |

| | | | | | | | | | | | | | | |
|---|---|---|---|---|---|---|---|---|---|---|---|---|---|---|
| Collin (2016)[86] | 316 | ED | Retrospective | Consecutive | Clear history of trauma to hip, negative or inconclusive conventional radiograph reports. | Not mentioned | MRI | 1.5 T Siemens Symphony whole-body scanner (Siemens Healthcare, Erlangen, Germany). | Clinical follow-up | Not mentioned | Female: 82, Male: 78 | 64.2% Female | Not mentioned | Not mentioned |
| Rehman (2016)[87] | 177 | ED | Retrospective | Not mentioned | Patients with suspected hip fractures and negative radiographs. | The patient received both CT and MRI | CT or MRI | Not mentioned | Clinical follow-up | Not mentioned | 82 (± 13) | 64.5% Female | Not mentioned | Not mentioned |
| Sadozai (2016)[88] | 78 | Royal Bolton Hospital | Retrospective | Not mentioned | All patients who underwent CT scanning for possible traumatic hip fractures | Any patients sustaining a periprosthetic fracture were excluded. | CT | Not mentioned | Clinical follow-up with MRI being used for two cases) | Not mentioned | Not mentioned | Not mentioned | Not mentioned | Not mentioned |
| Thomas (2016)[89] | 199 | University Hospital of Wales | Retrospective | Consecutive | Not mentioned | Not mentioned | CT | GE Lightspeed VCT 64-slice scanner | Clinical follow-up & MRI in four patients | GE 1.5-T Echospeed MR | Female: 83, Male: 84 | 68% Female | Not mentioned | Not mentioned |
| Lakshmanan (2017)[90] | 106 | Not mentioned | Retrospective | Consecutive | non-weight bearing with pain in the hip following a low-energy fall | Patients without a history of a fall and those with a history of more than six weeks of pain were excluded. Those with a history of trauma were also excluded. | MRI | 1.5T Excite HD (High Definition) (Signa; GE Medical Systems, Milwaukee, Wisconsin) with an eight-channel body coil | Clinical follow-up / Medical Record | Not mentioned | 81.4 | 56.6% Female | Not mentioned | Not mentioned |
| Lord (2017)[91] | 49 | Trauma Center | Retrospective | Consecutive | Patients undergoing MRI scans for acute hip pain with negative plain radiographs | Not mentioned | MRI | Not mentioned | Clinical follow-up | Not mentioned | 78 | 75% Female | Not mentioned | Not mentioned |
| Mandell (2018)[92] | 74 | ED | Retrospective | Consecutive | Clinical concern of hip fracture | Orthopaedic hardware in the affected hip, CT performed for any reason other than suspected hip or pelvic fracture (e.g., concern for infection or tumour), CT performed without radiographs, CT performed greater than 24 h after radiographs, or CT performed | CT | Siemens SOMATOM Definition Flash 128-slice dual-energy scanner & Siemens SOMATOM Definition AS+ 128-slice single-energy scanner | Surgical reports, MRI reports, and clinical follow-up | Not mentioned | 73 | 73% Female | Not mentioned | Not mentioned |

| | | | | | | | | | | | | | | |
|---|---|---|---|---|---|---|---|---|---|---|---|---|---|---|
| | | | | | | after radiographs identify a fracture | | | | | | | | |
| Ross (2019)[93] | 111 | ED | Retrospective | Consecutive | 65 years or older with clinically suspected hip fracture despite negative or equivocal radiographs who received a subsequent bony pelvis MRI within 48 h of initial evaluation | standard MRI protocol was not obtained or if there was metal hardware at the symptomatic hip | MRI | Not mentioned | Clinical follow-up in 30 days | Not Mentioned | 80 | 75% Female | Not mentioned | Not mentioned |
| Heynen (2019)[94] | 22 | ED | Prospective | Not mentioned | Patients over 50 years old were admitted to the ER for clinical suspicion of fracture of the hip or pelvis after low-energy trauma, Patients with negative or non-conclusive radiographs, and patients with suspected ROF and MDCT. | Evidence of fracture on radiographs, low clinical suspicion of fracture, Refusal to participate, contradiction to MRI, Incomplete MR exam, MR Scanner not available. | CT | (IQon Spectral CT®, Philips | MRI: The presence or absence of acute fracture was based on the retrospective analysis performed by a musculoskeletal radiologist with 35 years of experience in musculoskeletal imaging who had access to all medical and available imaging data including MDCT and MR images in the transverse plane | Magnetom Skyra®, Siemens Healthineers or Ingenia Achiva®, Philipsecified | 80.9 (± 12.5) | 59% Female | Not mentioned | A poorly defined medullary area that demonstrated decreased signal intensity on fat-sensitive images (T1-and T2-weighted Dixon fat-only images) and high signal intensity on fluid-sensitive sequences (STIR and T2-weighted Dixon water-only images) with or without a linear component within the marrow changes |
| Haims (2020)[95] | 81 | ED | Prospective | Not Specified | Negative X-ray within 24 hours before CT | Patient with hardware in the region of interest. | CT | General Electric (HD Discovery 64 slice or GE Revolution 64 Slice) or Siemens (Somatom Force or Somatom Definition Edge) scanners | Follow-up x-rays, MRI, and clinical examination | Not mentioned | Not mentioned | Not mentioned | Not mentioned | Presence of fracture on follow-up imaging |
| Lanotte (2019)[96] | 102 | ED | Retrospective | Consecutive | (a) age at least 60 years, (b) recent history of low-energy trauma, (c) clinical suspicion of femoral injury, and (d) normal or | Recent bone fracture on the pelvic and hip radiographs | CT | Not Specified | MRI | 3T Verio (Siemens Healthcare), 1.5T Avanto (Siemens Healthcare), 1.5T Optima MR450w | Male : 84.5 (± 8.3) ; Female : 82.8 (± 9.1) | 67.6% Female | Radiographs were considered to be normal in the absence of bone and joint | Not mentioned |

| | | | | | | | | | | | | | | |
|---|---|---|---|---|---|---|---|---|---|---|---|---|---|---|
| | | | | | non-contributive pelvic and hip radiographs | | | | | (General Electric) | | | disorders and non-contributive in the presence of radiographic changes unlikely to account for the patient complaints or in the case of hesitation about the likelihood of a fracture on the available radiographs | |
| Kutaiba (2020)[97] | 23 | ED | Retrospective | Not mentioned | Suspected NOF Fractures, negative or equivocal radiographs | Under 18 years old, Visible fractures on plain radiographs, the purpose of operative planning or other indication such as soft tissue injury, metastatic disease, prosthetic hip joints. | CT | 64-slice scanner (Revolution EVO; GE Healthcare, Chicago, IL, USA) ; 1.5T Avanto or 3T Skyra system (Siemens Healthcare, Erlangen, Germany) | MRI | 1.5T Avanto or 3T Skyra system (Siemens Healthcare, Erlangen, Germany) | 82 | 60.8% Female | Not mentioned | Not mentioned |
| Tsukamoto (2023)[98] | 94 | ED | Retrospective | Not mentioned | Symptoms of groin pain with the following 3 findings were judged as hip pain in this population: tenderness of the Scarpa triangle, groin pain during weight bearing, and groin pain when moving the hip joint; >70 years old. | high energy trauma or fever. Rheumatoid arthritis, pyogenic arthritis, excess alcohol consumption, and steroid use. | US | Sonimage HS1 device (Konica Minolta, Inc., Tokyo, Japan) | MRI | Not mentioned | 81.8 | 88.2% Female | Not mentioned | Not mentioned |
| Reddy (2015)[99] | 25 | ED | Retrospective | Consecutive | Suspicion of Hip fracture & normal radiographs | Patients with orthopaedic hardware | CT (DECT with VNC) | Definition FLASH, Siemens, Erlangen, Germany) | Clinical and radiograph follow-up | Not specified | 77 | 72% Female | Not mentioned | Not mentioned |

ED; Emergency department, MRI, Magnetic resonance imaging, CT, Computed tomography; N/A, Not applicable; US, Ultrasonography; MDCT, Multidetector computed tomography; STIR, Short inversion tau recovery; NOF, Non-occult fracture; ROF, Radiographically occult fracture

Supplementary Table S8. Notable exclusions for the hip fracture

| Author (Year) | Reason |
|---|---|
| Davidson (2021)(100) | Not all patients underwent MRI, hence a 2x2 table information could not be extracted reliably |
| Ozimok (2020)(101) | The reference test does not fulfil our criteria |
| Bin (2022)(102) | Not all patients are negative for radiograph fractures |
| Haris (2019)(103) | Not all patients are negative for radiograph fractures |
| Avci (2019)(104) | Not all patients are negative for radiograph fractures |
| Eggenberger (2019)(105) | Not all patients are negative for radiograph fractures |
| Lee (2018)(106) | No reference test was conducted |
| Akgun (2019)(107) | No reference test was conducted |
| Moon (2018)(108) | Initial radiographs are positive for fractures |
| Gatt (2021)(109) | No reference test was conducted |
| Sharrock (2022)(110) | No reference test was conducted |
| Kim (2013)(111) | Not all patients underwent the reference test |
| Lee (2010)(112) | Not all patients are negative for radiograph fractures, only the extensions are occult. |
| Guanche (1994)(113) | No reference test was conducted |
| Collin (2016)(114) | Deemed to be the same cohort as Collin (2016)(86) |
| Collin (2016)(115) | |

MRI; Magnetic resonance imaging

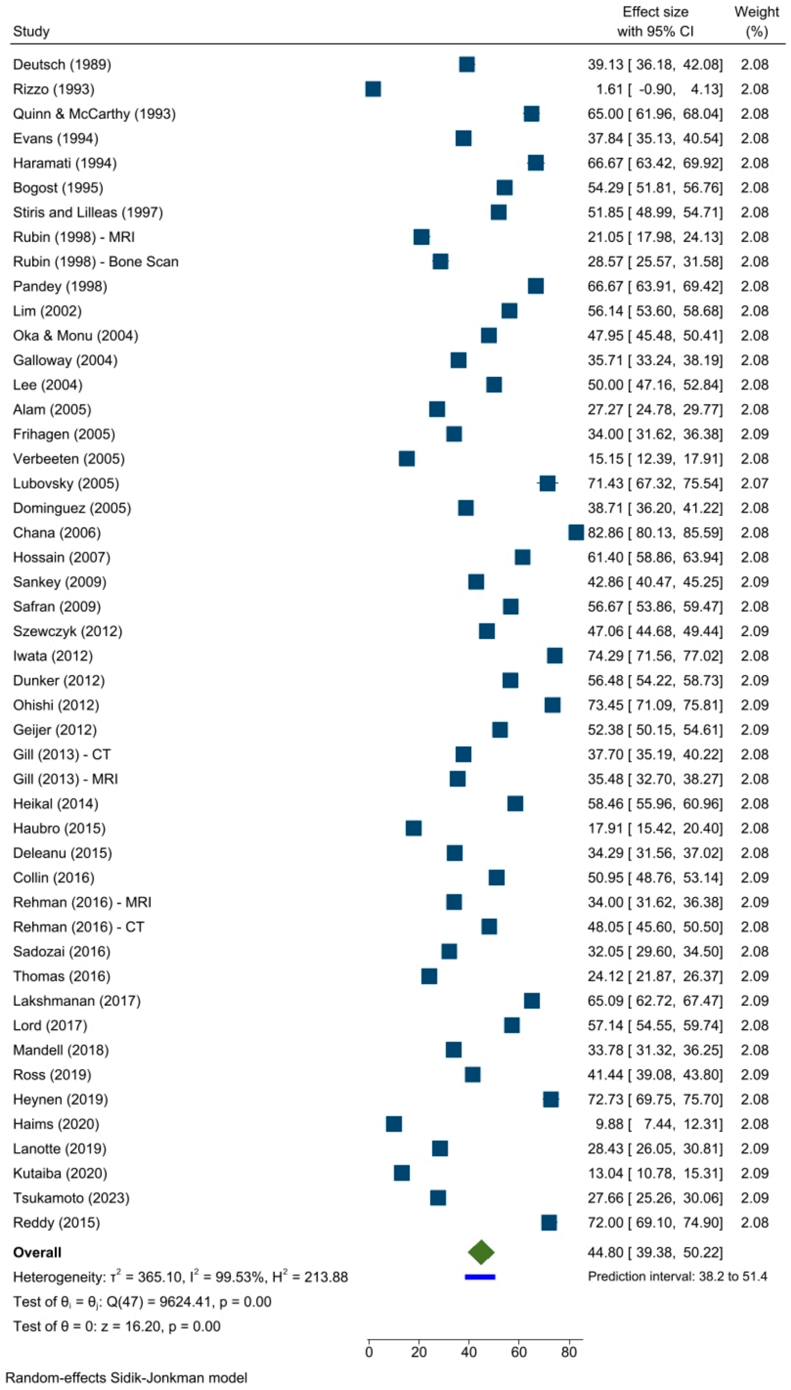


Supplementary Figure S11. Meta-Analysis of Prevalence of Occult Hip and Femoral Fracture

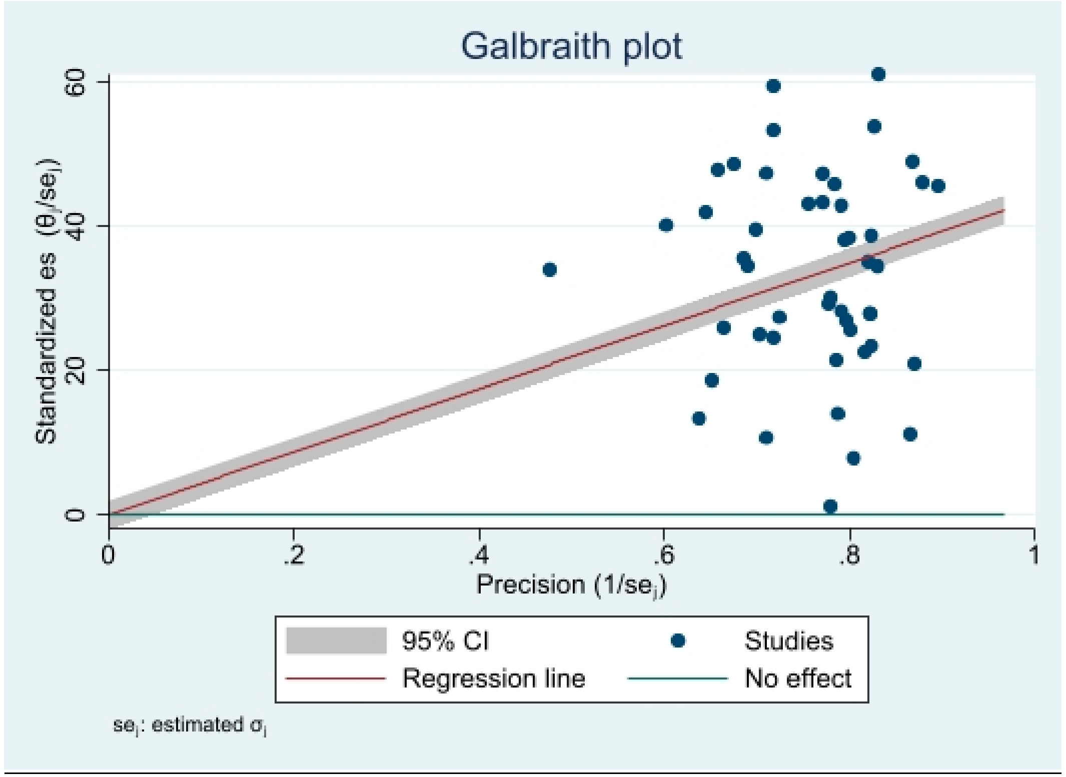


Supplementary Figure S12. Galbraih plot of occult scaphoid fracture

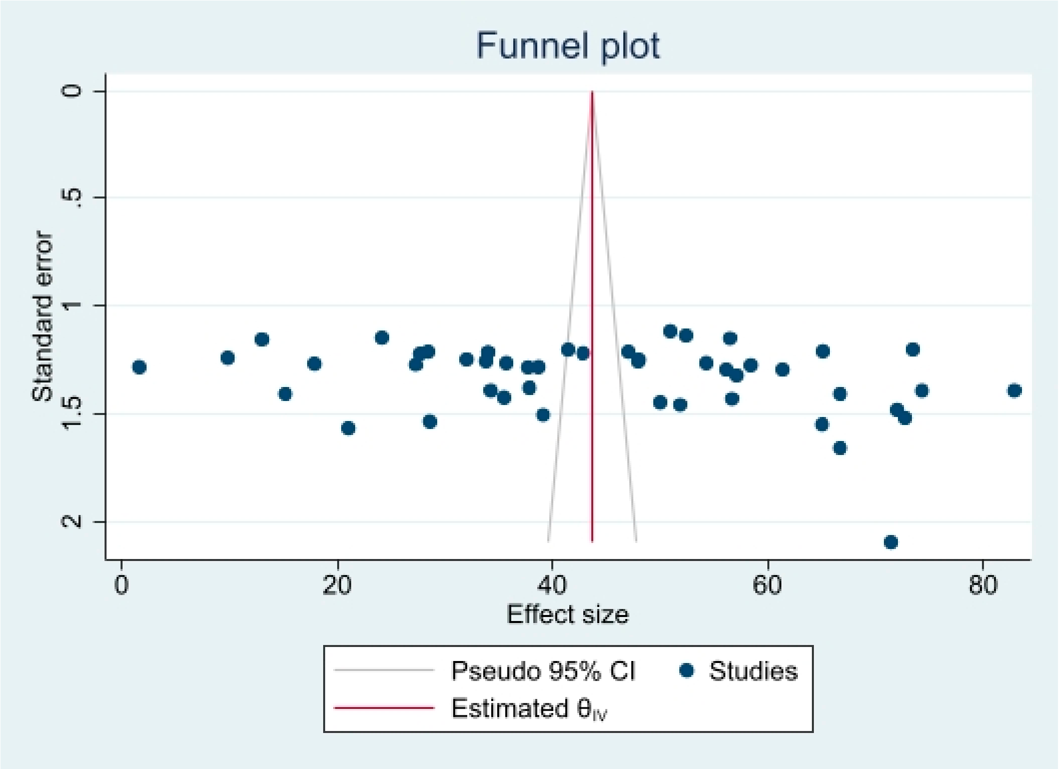

Supplementary Figure S13. Funnel plot of studies included in the prevalence of occult hip and femoral fracture

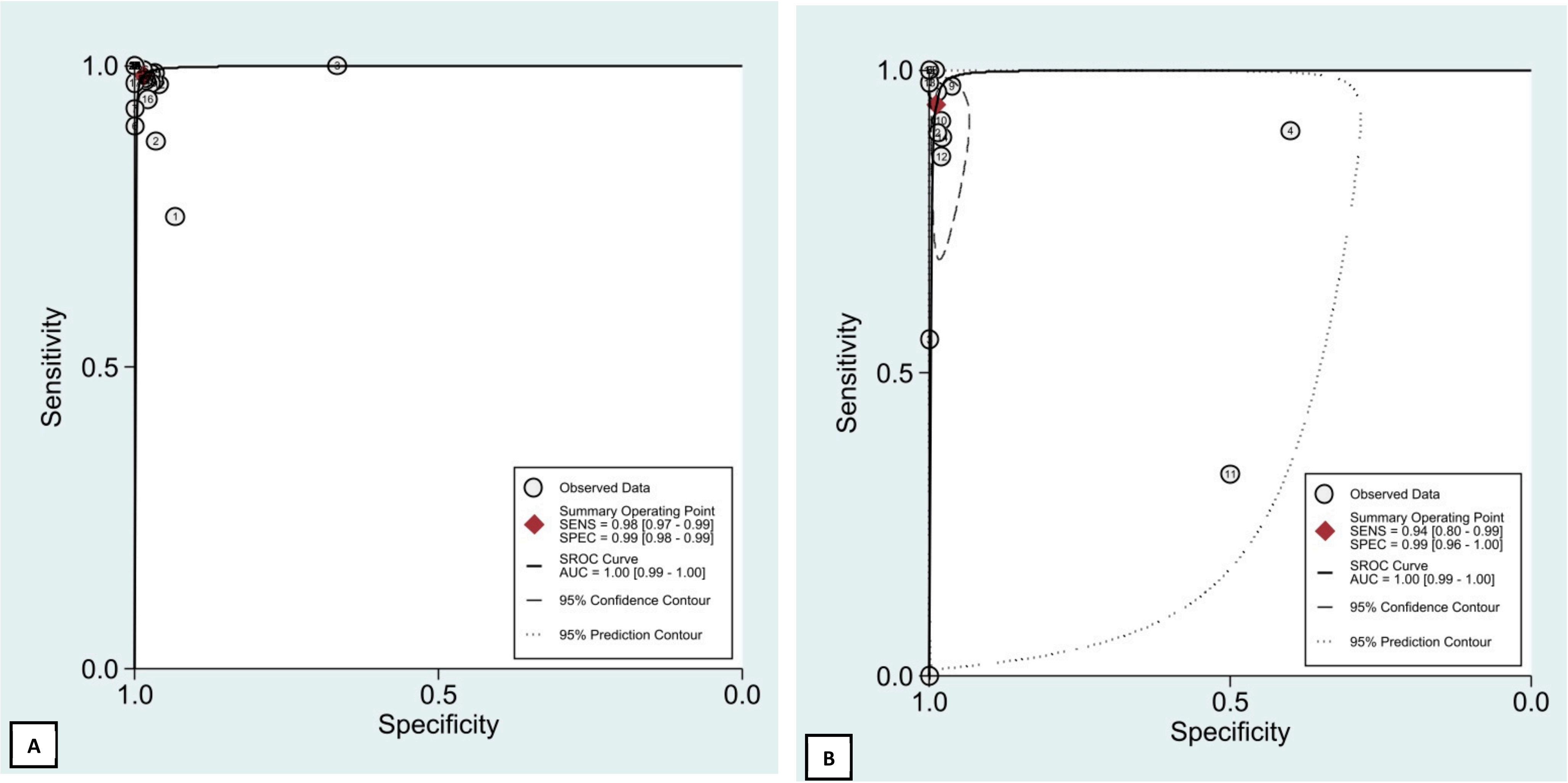


Supplementary Figure S14. The hierarchical summary receiver operating characteristic (HSROC) of CT (A) and MRI (B) in detecting occult hip and femoral fracture

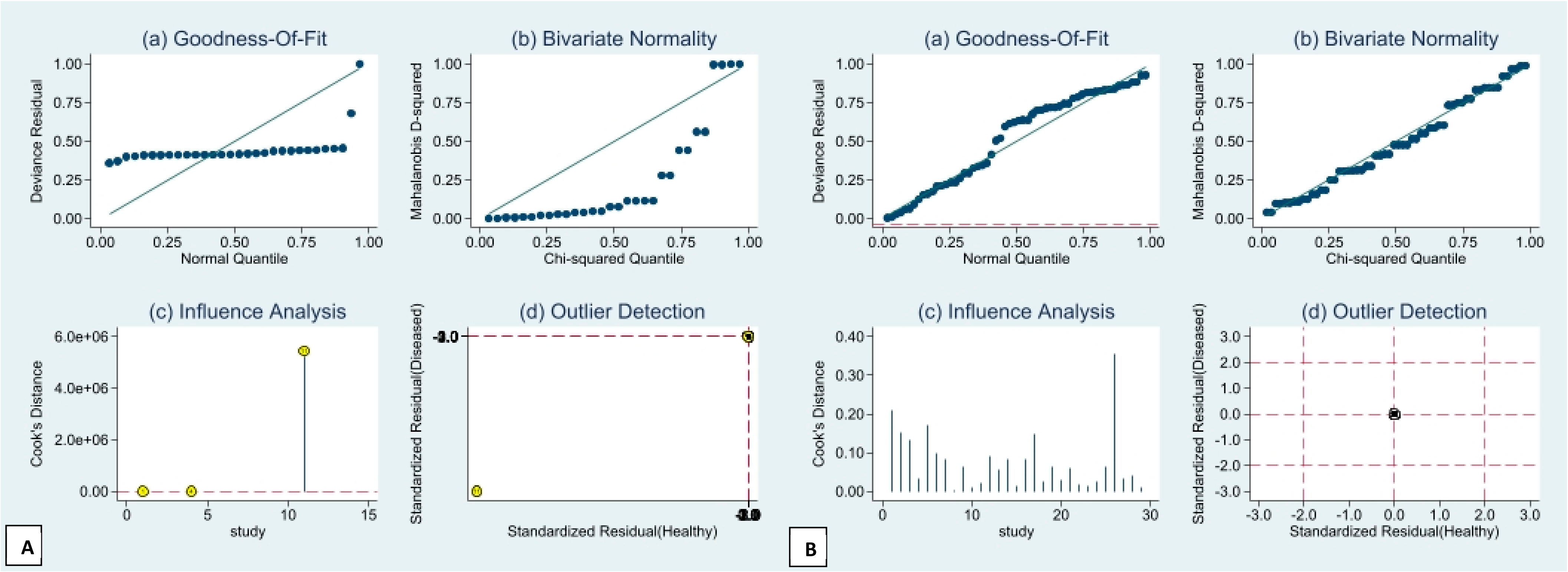


Supplementary Figure S15. Model diagnostics of each study for CT (A) and MRI (B) in detecting occult hip and femoral fracture

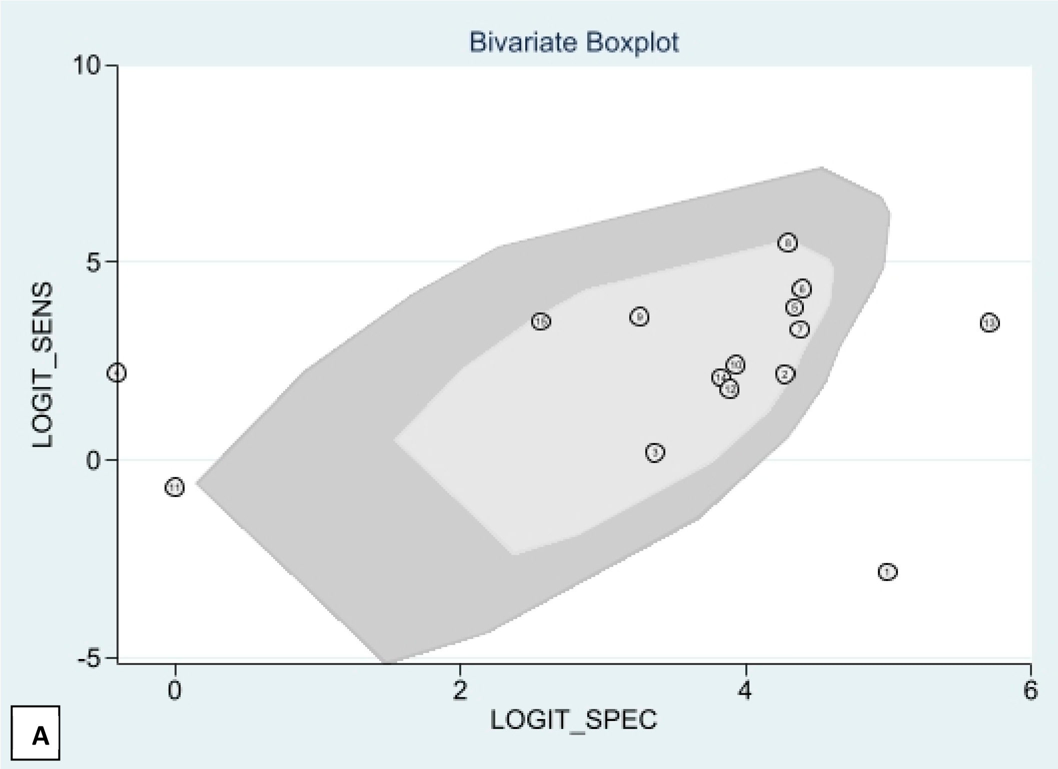


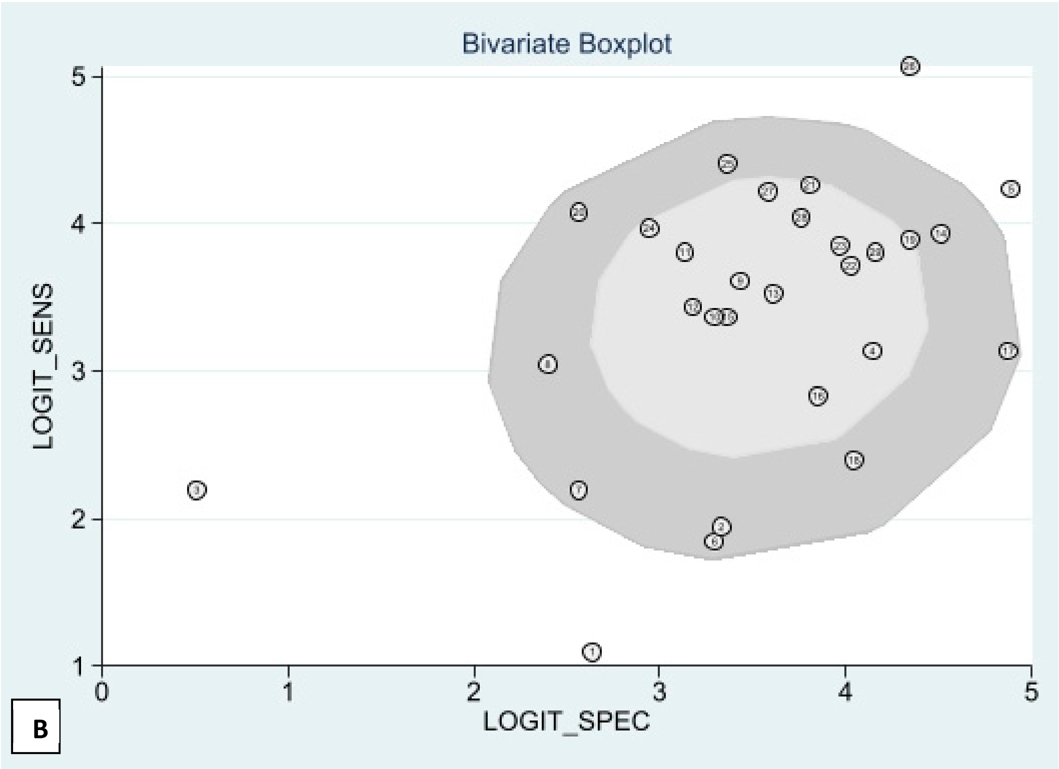


Supplementary Figure S16. Bivariate boxplot of each study for CT (A) and MRI (B) in detecting occult hip and femoral fracture

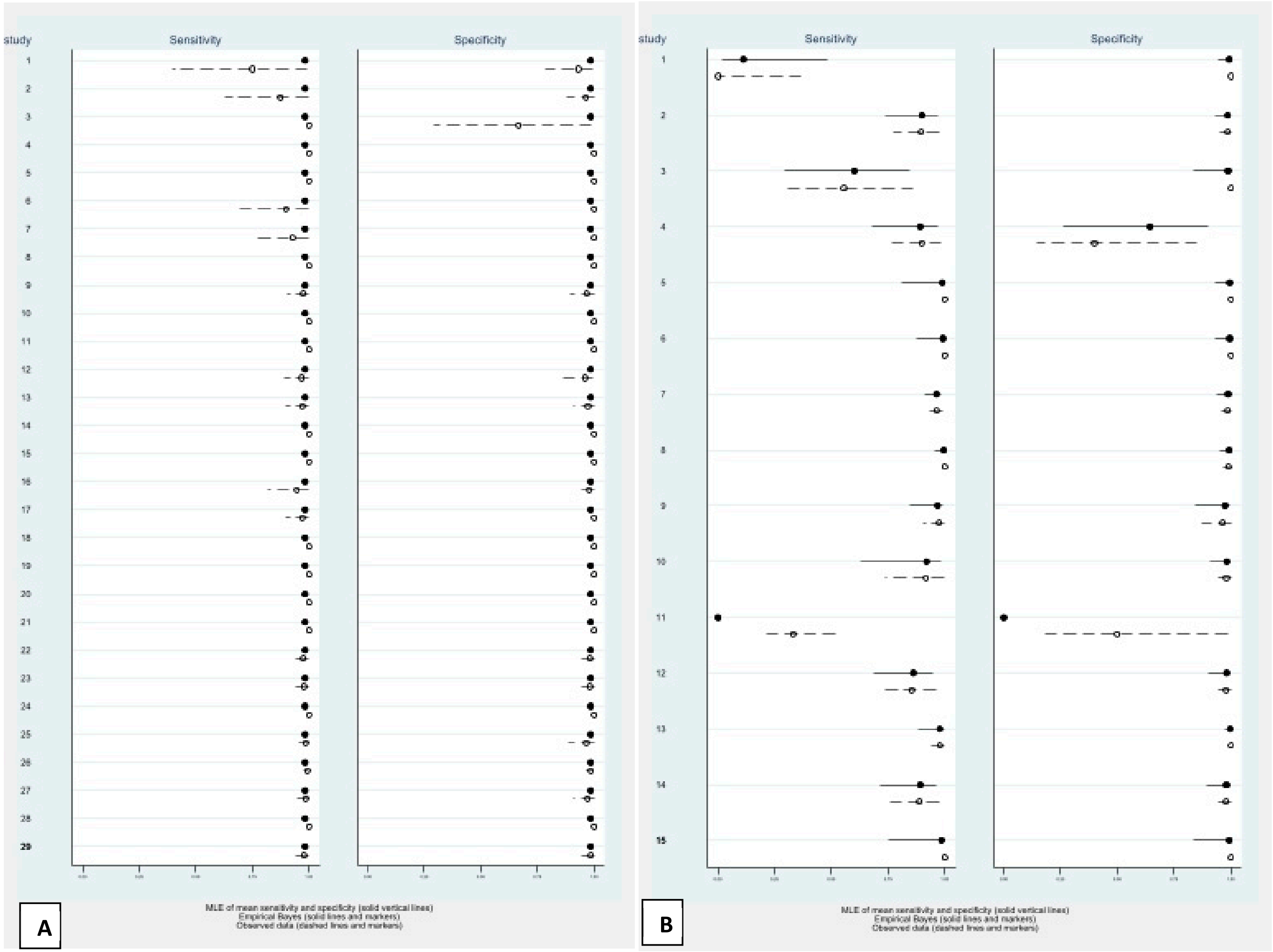


Supplementary Figure S17. Empirical Bayes prediction of sensitivity and specificity of each study for CT (A) and MRI (B) in detecting occult hip and femoral fracture

Supplementary References


1. Yang TW, Lin YY, Hsu SC, Chu KC, Hsiao CW, Hsu CW, et al. Diagnostic performance of cone-beam computed tomography for scaphoid fractures: a systematic review and diagnostic meta-analysis. Sci Rep. 2021;11(1):2587.
2. Neubauer J, Benndorf M, Ehritt-Braun C, Reising K, Yilmaz T, Klein C, et al. Comparison of the diagnostic accuracy of cone beam computed tomography and radiography for scaphoid fractures. Scientific Reports. 2018;8(1):3906.
3. Gibney B, Smith M, Moughty A, Kavanagh EC, Hynes D, MacMahon PJ. Incorporating Cone-Beam CT Into the Diagnostic Algorithm for Suspected Radiocarpal Fractures: A New Standard of Care? AJR Am J Roentgenol. 2019;213(5):1117-23.
4. Najaf-Zadeh A, Nectoux E, Dubos F, Happiette L, Demondion X, Gnansounou M, et al. Prevalence and clinical significance of occult fractures in children with radiograph-negative acute ankle injury. A meta-analysis. Acta Orthop. 2014;85(5):518-24.
5. Joshi N, Lira A, Mehta N, Paladino L, Sinert R. Diagnostic accuracy of history, physical examination, and bedside ultrasound for diagnosis of extremity fractures in the emergency department: a systematic review. Acad Emerg Med. 2013;20(1):1-15.
6. Ghazi Sherbaf F, Sair HI, Shakoor D, Fritz J, Schwaiger BJ, Johnson MH, et al. DECT in Detection of Vertebral Fracture-associated Bone Marrow Edema: A Systematic Review and Meta-Analysis with Emphasis on Technical and Imaging Interpretation Parameters. Radiology. 2021;300(1):110-9.
7. Gilbertson J, Pageau P, Ritcey B, Cheng W, Burwash-Brennan T, Perry JJ, et al. Test Characteristics of Chest Ultrasonography for Rib Fractures Following Blunt Chest Trauma: A Systematic Review and Meta-analysis. Ann Emerg Med. 2022;79(6):529-39.
8. Adeyemo WL, Akadiri OA. A systematic review of the diagnostic role of ultrasonography in maxillofacial fractures. Int J Oral Maxillofac Surg. 2011;40(7):655-61.
9. Fitzpatrick E, Sharma V, Rojoa D, Raheman F, Singh H. The use of cone-beam computed tomography (CBCT) in radiocarpal fractures: a diagnostic test accuracy meta-analysis. Skeletal Radiol. 2022;51(5):923-34.
10. Luís Duarte M, Dos Santos LR, Oliveira ASB, Iared W, Peccin MS. Computed tomography with low-dose radiation versus standard-dose radiation for diagnosing fractures: systematic review and meta-analysis. Sao Paulo Med J. 2021;139(4):388-97.
11. Yin ZG, Zhang JB, Kan SL, Wang XG. Diagnosing suspected scaphoid fractures: a systematic review and meta-analysis. Clin Orthop Relat Res. 2010;468(3):723-34.
12. Offiah AC, Burke D. The diagnostic accuracy of cross-sectional imaging for detecting acute scaphoid fractures in children: a systematic review. Br J Radiol. 2018;91(1086):20170883.
13. Champagne N, Eadie L, Regan L, Wilson P. The effectiveness of ultrasound in the detection of fractures in adults with suspected upper or lower limb injury: a systematic review and subgroup meta-analysis. BMC Emerg Med. 2019;19(1):17.
14. Wu J, Wang Y, Wang Z. The diagnostic accuracy of ultrasound in the detection of foot and ankle fractures: a systematic review and meta-analysis. Med Ultrason. 2021;23(2):203-12.
15. Yin ZG, Zhang JB, Kan SL, Wang XG. Diagnostic accuracy of imaging modalities for suspected scaphoid fractures: meta-analysis combined with latent class analysis. J Bone Joint Surg Br. 2012;94(8):1077-85.
16. Alabousi M, Gauthier ID, Li N, Dos Santos GM, Golev D, Patlas MN, et al. Multi-detector CT for suspected hip fragility fractures: A diagnostic test accuracy systematic review and meta-analysis. Emerg Radiol. 2019;26(5):549-56.
17. Gordon I, Sinert R, Chao J. The Utility of Ultrasound in Detecting Skull Fractures After Pediatric Blunt Head Trauma: Systematic Review and Meta-Analysis. Pediatr Emerg Care. 2021;37(12):e1701-e7.
18. Mennen AHM, Blokland AS, Maas M, van Embden D. Imaging of pelvic ring fractures in older adults and its clinical implications-a systematic review. Osteoporos Int. 2023;34(9):1549-59.
19. Gadicherla S, Pentapati KC, Rustaqi N, Singh A, Smriti K. Diagnostic Accuracy of Ultrasonography for the Assessment of Maxillofacial Fractures: A Meta-analysis. J Int Soc Prev Community Dent. 2021;11(5):503-9.

20. Alexandridis G, Verschuuren EW, Rosendaal AV, Kanhai DA. Evidence base for point-of-care ultrasound (POCUS) for diagnosis of skull fractures in children: a systematic review and meta-analysis. Emerg Med J. 2022;39(1):30-6.
21. Malgo F, Hamdy NAT, Ticheler C, Smit F, Kroon HM, Rabelink TJ, et al. Value and potential limitations of vertebral fracture assessment (VFA) compared to conventional spine radiography: experience from a fracture liaison service (FLS) and a meta-analysis. Osteoporos Int. 2017;28(10):2955-65.
22. Jain R, Jain N, Sheikh T, Yadav C. Early scaphoid fractures are better diagnosed with ultrasonography than X-rays: A prospective study over 114 patients. Chin J Traumatol. 2018;21(4):206-10.
23. Nguyen JC, Shah AS, Nguyen MK, Baghdadi S, Nicholson A, Guariento A, et al. Pediatric scaphoid fracture: diagnostic performance of various radiographic views. Emerg Radiol. 2021;28(3):565-72.
24. Daniels AM, Kranendonk J, Wyers CE, Janzing HMJ, Sassen S, van Rietbergen B, et al. What Is the Diagnostic Performance of Conventional Radiographs and Clinical Reassessment Compared With HR-pQCT Scaphoid Fracture Diagnosis? Clin Orthop Relat Res. 2023;481(1):97-104.
25. Bulstra AEJ, van Boxel MF, Crijns TJ, Kelly J, Obdeijn MC, Kerkhoffs G, et al. Routine MRI Among Patients With a Suspected Scaphoid Fracture Risks Overdiagnosis. Clin Orthop Relat Res. 2023;481(12):2309-15.
26. Dean BJF, Little C, Riley ND, Sellon E, Sheehan W, Burford J, et al. Suspected scaphoid injuries managed by MRI direct from the emergency department : a single-centre prospective cohort study. Bone Jt Open. 2021;2(6):447-53.
27. Etli I, Kozaci N, Avci M, Karakoyun OF. Comparison of the diagnostic accuracy of X-ray and computed tomography in patients with wrist injury. Injury. 2020;51(3):651-5.
28. Kim JE, Yoo HJ, Chae HD, Choi JY, Hong SH, Kang JH, et al. Dual-Layer Detector CT With Virtual Noncalcium Imaging: Diagnostic Performance in Patients With Suspected Wrist Fractures. AJR Am J Roentgenol. 2021;216(4):1003-13.
29. Kelson T, Davidson R, Baker T. Early MRI versus conventional management in the detection of occult scaphoid fractures: what does it really cost? A rural pilot study. J Med Radiat Sci. 2016;63(1):9-16.
30. Rolfe EB, Garvie NW, Khan MA, Ackery DM. Isotope bone imaging in suspected scaphoid trauma. Br J Radiol. 1981;54(645):762-7.
31. Wilson AW, Kurer MH, Peggington JL, Grant DS, Kirk CC. Bone scintigraphy in the management of X-ray-negative potential scaphoid fractures. Arch Emerg Med. 1986;3(4):235-42.
32. Yıldırım A, Unlüer EE, Vandenberk N, Karagöz A. The role of bedside ultrasonography for occult scaphoid fractures in the emergency department. Ulus Travma Acil Cerrahi Derg. 2013;19(3):241-5.
33. Platon A, Poletti PA, Van Aaken J, Fusetti C, Della Santa D, Beaulieu JY, et al. Occult fractures of the scaphoid: the role of ultrasonography in the emergency department. Skeletal Radiol. 2011;40(7):869-75.
34. Fusetti C, Poletti PA, Pradel PH, Garavaglia G, Platon A, Della Santa DR, et al. Diagnosis of occult scaphoid fracture with high-spatial-resolution sonography: a prospective blind study. J Trauma. 2005;59(3):677-81.
35. Senall JA, Failla JM, Bouffard JA, van Holsbeeck M. Ultrasound for the early diagnosis of clinically suspected scaphoid fracture. J Hand Surg Am. 2004;29(3):400-5.
36. Hauger O, Bonnefoy O, Moinard M, Bersani D, Diard F. Occult fractures of the waist of the scaphoid: early diagnosis by high-spatial-resolution sonography. AJR Am J Roentgenol. 2002;178(5):1239-45.
37. Herneth AM, Siegmeth A, Bader TR, Ba-Ssalamah A, Lechner G, Metz VM, et al. Scaphoid fractures: evaluation with high-spatial-resolution US initial results. Radiology. 2001;220(1):231-5.
38. Xie C, Ather S, Mansour R, Gleeson F, Chowdhury R. Dual-energy CT in the diagnosis of occult acute scaphoid injury: a direct comparison with MRI. Eur Radiol. 2021;31(6):3610-5.
39. Kitsis C, Taylor M, Chandey J, Smith R, Latham J, Turner S, et al. Imaging the problem scaphoid. Injury. 1998;29(7):515-20.

40. Thorpe AP, Murray AD, Smith FW, Ferguson J. Clinically suspected scaphoid fracture: a comparison of magnetic resonance imaging and bone scintigraphy. Br J Radiol. 1996;69(818):109-13.
41. Fowler C, Sullivan B, Williams LA, McCarthy G, Savage R, Palmer A. A comparison of bone scintigraphy and MRI in the early diagnosis of the occult scaphoid waist fracture. Skeletal Radiol. 1998;27(12):683-7.
42. Borel C, Larbi A, Delclaux S, Lapegue F, Chiavassa-Gandois H, Sans N, et al. Diagnostic value of cone beam computed tomography (CBCT) in occult scaphoid and wrist fractures. Eur J Radiol. 2017;97:59-64.
43. Edlund R, Skorpil M, Lapidus G, Bäcklund J. Cone-Beam CT in diagnosis of scaphoid fractures. Skeletal Radiol. 2016;45(2):197-204.
44. de Zwart A, Rhemrev SJ, Kingma LM, Meylaerts SA, Arndt JW, Schipper IB, et al. Early CT compared with bone scintigraphy in suspected schapoid fractures. Clin Nucl Med. 2012;37(10):981.
45. Beeres FJ, Rhemrev SJ, den Hollander P, Kingma LM, Meylaerts SA, le Cessie S, et al. Early magnetic resonance imaging compared with bone scintigraphy in suspected scaphoid fractures. J Bone Joint Surg Br. 2008;90(9):1205-9.
46. Ilica AT, Ozyurek S, Kose O, Durusu M. Diagnostic accuracy of multidetector computed tomography for patients with suspected scaphoid fractures and negative radiographic examinations. Jpn J Radiol. 2011;29(2):98-103.
47. Mallee W, Doornberg JN, Ring D, van Dijk CN, Maas M, Goslings JC. Comparison of CT and MRI for diagnosis of suspected scaphoid fractures. J Bone Joint Surg Am. 2011;93(1):20-8.
48. Memarsadeghi M, Breitenseher MJ, Schaefer-Prokop C, Weber M, Aldrian S, Gäbler C, et al. Occult scaphoid fractures: comparison of multidetector CT and MR imaging--initial experience. Radiology. 2006;240(1):169-76.
49. Breitenseher MJ, Metz VM, Gilula LA, Gaebler C, Kukla C, Fleischmann D, et al. Radiographically occult scaphoid fractures: value of MR imaging in detection. Radiology. 1997;203(1):245-50.
50. Tiel-van Buul MM, Roolker W, Verbeeten BW, Broekhuizen AH. Magnetic resonance imaging versus bone scintigraphy in suspected scaphoid fracture. Eur J Nucl Med. 1996;23(8):971-5.
51. Nielsen PT, Hedeboe J, Thommesen P. Bone scintigraphy in the evaluation of fracture of the carpal scaphoid bone. Acta Orthop Scand. 1983;54(2):303-6.
52. O'Carroll PF, Doyle J, Duffy G. Radiography and scintigraphy in the diagnosis of carpal scaphoid fractures. Ir J Med Sci. 1982;151(7):211-3.
53. Stordahl A, Schjøth A, Woxholt G, Fjermeros H. Bone scanning of fractures of the scaphoid. J Hand Surg Br. 1984;9(2):189-90.
54. Tiel-van Buul MM, van Beek EJ, Broekhuizen AH, Bakker AJ, Bos KE, van Royen EA. Radiography and scintigraphy of suspected scaphoid fracture. A long-term study in 160 patients. J Bone Joint Surg Br. 1993;75(1):61-5.
55. Deutsch AL, Mink JH, Waxman AD. Occult fractures of the proximal femur: MR imaging. Radiology. 1989;170(1 Pt 1):113-6.
56. Rizzo PF, Gould ES, Lyden JP, Asnis SE. Diagnosis of occult fractures about the hip. Magnetic resonance imaging compared with bone-scanning. JBJS. 1993;75(3):395-401.
57. Quinn SF, McCarthy JL. Prospective evaluation of patients with suspected hip fracture and indeterminate radiographs: use of T1-weighted MR images. Radiology. 1993;187(2):469-71.
58. Evans PD, Wilson C, Lyons K. Comparison of MRI with bone scanning for suspected hip fracture in elderly patients. J Bone Joint Surg Br. 1994;76(1):158-9.
59. Haramati N, Staron RB, Barax C, Feldman F. Magnetic resonance imaging of occult fractures of the proximal femur. Skeletal Radiol. 1994;23(1):19-22.
60. Bogost GA, Lizerbram EK, Crues JV, 3rd. MR imaging in evaluation of suspected hip fracture: frequency of unsuspected bone and soft-tissue injury. Radiology. 1995;197(1):263-7.
61. Stiris MG, Lilleås FG. MR findings in cases of suspected impacted fracture of the femoral neck. Acta Radiol. 1997;38(5):863-6.
62. Rubin SJ, Marquardt JD, Gottlieb RH, Meyers SP, Totterman SM, O'Mara RE. Magnetic resonance imaging: a cost-effective alternative to bone scintigraphy in the evaluation of patients with suspected hip fractures. Skeletal Radiol. 1998;27(4):199-204.

63. Pandey R, McNally E, Ali A, Bulstrode C. The role of MRI in the diagnosis of occult hip fractures. Injury. 1998;29(1):61-3.
64. Lim KB, Eng AK, Chng SM, Tan AG, Thoo FL, Low CO. Limited magnetic resonance imaging (MRI) and the occult hip fracture. Ann Acad Med Singap. 2002;31(5):607-10.
65. Oka M, Monu JU. Prevalence and patterns of occult hip fractures and mimics revealed by MRI. AJR Am J Roentgenol. 2004;182(2):283-8.
66. Galloway HR, Meikle GR, Despois M. Patterns of injury in patients with radiographic occult fracture of neck of femur as determined by magnetic resonance imaging. Australas Radiol. 2004;48(1):21-4.
67. Lee YP, Griffith JF, Antonio GE, Tang N, Leung KS. Early magnetic resonance imaging of radiographically occult osteoporotic fractures of the femoral neck. Hong Kong Med J. 2004;10(4):271-5.
68. Alam A, Willett K, Ostlere S. The MRI diagnosis and management of incomplete intertrochanteric fractures of the femur. J Bone Joint Surg Br. 2005;87(9):1253-5.
69. Frihagen F, Nordsletten L, Tariq R, Madsen JE. MRI diagnosis of occult hip fractures. Acta Orthop. 2005;76(4):524-30.
70. Verbeeten KM, Hermann KL, Hasselqvist M, Lausten GS, Joergensen P, Jensen CM, et al. The advantages of MRI in the detection of occult hip fractures. Eur Radiol. 2005;15(1):165-9.
71. Lubovsky O, Liebergall M, Mattan Y, Weil Y, Mosheiff R. Early diagnosis of occult hip fractures MRI versus CT scan. Injury. 2005;36(6):788-92.
72. Dominguez S, Liu P, Roberts C, Mandell M, Richman PB. Prevalence of traumatic hip and pelvic fractures in patients with suspected hip fracture and negative initial standard radiographs--a study of emergency department patients. Acad Emerg Med. 2005;12(4):366-9.
73. Chana R, Noorani A, Ashwood N, Chatterji U, Healy J, Baird P. The role of MRI in the diagnosis of proximal femoral fractures in the elderly. Injury. 2006;37(2):185-9.
74. Hossain M, Barwick C, Sinha AK, Andrew JG. Is magnetic resonance imaging (MRI) necessary to exclude occult hip fracture? Injury. 2007;38(10):1204-8.
75. Sankey RA, Turner J, Lee J, Healy J, Gibbons CE. The use of MRI to detect occult fractures of the proximal femur: a study of 102 consecutive cases over a ten-year period. J Bone Joint Surg Br. 2009;91(8):1064-8.
76. Safran O, Goldman V, Applbaum Y, Milgrom C, Bloom R, Peyser A, et al. Posttraumatic painful hip: sonography as a screening test for occult hip fractures. J Ultrasound Med. 2009;28(11):1447-52.
77. Szewczyk-Bieda M, Thomas N, Oliver TB. Radiographically occult femoral and pelvic fractures are not mutually exclusive: a review of fractures detected by MRI following low-energy trauma. Skeletal Radiol. 2012;41(9):1127-32.
78. Iwata T, Nozawa S, Dohjima T, Yamamoto T, Ishimaru D, Tsugita M, et al. The value of T1-weighted coronal MRI scans in diagnosing occult fracture of the hip. J Bone Joint Surg Br. 2012;94(7):969-73.
79. Dunker D, Collin D, Göthlin JH, Geijer M. High clinical utility of computed tomography compared to radiography in elderly patients with occult hip fracture after low-energy trauma. Emerg Radiol. 2012;19(2):135-9.
80. Ohishi T, Ito T, Suzuki D, Banno T, Honda Y. Occult hip and pelvic fractures and accompanying muscle injuries around the hip. Arch Orthop Trauma Surg. 2012;132(1):105-12.
81. Geijer M, Dunker D, Collin D, Göthlin JH. Bone bruise, lipohemarthrosis, and joint effusion in CT of non-displaced hip fracture. Acta Radiol. 2012;53(2):197-202.
82. Gill SK, Smith J, Fox R, Chesser TJ. Investigation of occult hip fractures: the use of CT and MRI. ScientificWorldJournal. 2013;2013:830319.
83. Heikal S, Riou P, Jones L. The use of computed tomography in identifying radiologically occult hip fractures in the elderly. Ann R Coll Surg Engl. 2014;96(3):234-7.
84. Haubro M, Stougaard C, Torfing T, Overgaard S. Sensitivity and specificity of CT- and MRI-scanning in evaluation of occult fracture of the proximal femur. Injury. 2015;46(8):1557-61.
85. Deleanu B, Prejbeanu R, Tsiridis E, Vermesan D, Crisan D, Haragus H, et al. Occult fractures of the proximal femur: imaging diagnosis and management of 82 cases in a regional trauma center. World J Emerg Surg. 2015;10:55.
86. Collin D, Geijer M, Göthlin JH. Prevalence of exclusively and concomitant pelvic fractures at magnetic resonance imaging of suspect and occult hip fractures. Emerg Radiol. 2016;23(1):17-21.

87. Rehman H, Clement RG, Perks F, White TO. Imaging of occult hip fractures: CT or MRI? Injury. 2016;47(6):1297-301.
88. Sadozai Z, Davies R, Warner J. The sensitivity of ct scans in diagnosing occult femoral neck fractures. Injury. 2016;47(12):2769-71.
89. Thomas RW, Williams HL, Carpenter EC, Lyons K. The validity of investigating occult hip fractures using multidetector CT. Br J Radiol. 2016;89(1060):20150250.
90. Lakshmanan P, Sharma A, Lyons K, Peehal JP. Are occult fractures of the hip and pelvic ring mutually exclusive? J Bone Joint Surg Br. 2007;89(10):1344-6.
91. Lord C, Leach S, Skiadas VT, editors. MRI for suspected hip fracture: how many sequences are enough? ESSR 2017; 2017.
92. Mandell JC, Weaver MJ, Khurana B. Computed tomography for occult fractures of the proximal femur, pelvis, and sacrum in clinical practice: single institution, dual-site experience. Emerg Radiol. 2018;25(3):265-73.
93. Ross AB, Chan BY, Yi PH, Repplinger MD, Vanness DJ, Lee KS. Diagnostic accuracy of an abbreviated MRI protocol for detecting radiographically occult hip and pelvis fractures in the elderly. Skeletal Radiol. 2019;48(1):103-8.
94. Heynen B, Tamigneaux C, Pasoglou V, Malghem J, Vande Berg B, Kirchgesner T. MRI detection of radiographically occult fractures of the hip and pelvis in the elderly: Comparison of T2-weighted Dixon sequence with T1-weighted and STIR sequences. Diagn Interv Imaging. 2019;100(3):169-75.
95. Haims AH, Wang A, Yoo BJ, Porrino J. Negative predictive value of CT for occult fractures of the hip and pelvis with imaging follow-up. Emerg Radiol. 2021;28(2):259-64.
96. Lanotte SJ, Larbi A, Michoux N, Baron MP, Hamard A, Mourad C, et al. Value of CT to detect radiographically occult injuries of the proximal femur in elderly patients after low-energy trauma: determination of non-inferiority margins of CT in comparison with MRI. Eur Radiol. 2020;30(2):1113-26.
97. Kutaiba N, Lamanna A, Malara F, Yap LP. Use of computed tomography and magnetic resonance imaging for occult neck of femur fractures: A single-centre study. Emerg Med Australas. 2020;32(6):980-5.
98. Tsukamoto H, Kijima H, Saito K, Saito H, Miyakoshi N. Diagnostic accuracy of ultrasonography for occult femoral neck fracture. Journal of Clinical Orthopaedics and Trauma. 2023;36:102087.
99. Reddy T, McLaughlin PD, Mallinson PI, Reagan AC, Munk PL, Nicolaou S, et al. Detection of occult, undisplaced hip fractures with a dual-energy CT algorithm targeted to detection of bone marrow edema. Emerg Radiol. 2015;22(1):25-9.
100. Davidson A, Silver N, Cohen D, Gross M, Zinger G, Applbaum Y, et al. Justifying CT prior to MRI in cases of suspected occult hip fracture. A proposed diagnostic protocol. Injury. 2021;52(6):1429-33.
101. Ozimok C, Koff D, Parasu N. Emphasizing the Diagnostic Value of Digital Tomosynthesis in Detecting Hip Fractures. Tomography. 2020;6(3):308-14.
102. Bin C, Zhixin L, Hong'an T. MPR versus VR of multi-slice CT scans in diagnosis of subtle and occult fractures. International Medicine and Health Guidance News. 2022;28(7):950-3.
103. Haris M, Robinson P, Gupta H. Evaluation of Occult Femoral Neck Fractures – Computed Tomography or Magnetic Resonance Imaging? Indian Journal of Musculoskeletal Radiology. 2019;1.
104. Avci M, Kozaci N, Tulubas G, Caliskan G, Yuksel A, Karaca A, et al. Comparison of Point-of-Care Ultrasonography and Radiography in the Diagnosis of Long-Bone Fractures. Medicina. 2019;55(7):355.
105. Eggenberger E, Hildebrand G, Vang S, Ly A, Ward C. Use of CT Vs. MRI for Diagnosis of Hip or Pelvic Fractures in Elderly Patients After Low Energy Trauma. Iowa Orthop J. 2019;39(1):179-83.
106. Lee YK, Lee YJ, Lee NK, Nho JH, Koo KH. Low Positive Predictive Value of Bone Scan to Predict Impending Complete Fracture among Incomplete Atypical Femoral Fracture. J Korean Med Sci. 2018;33(22):e157.
107. Akgun U, Canbek U, Aydogan NH. Reliability and diagnostic utility of radiographs in patients with incomplete atypical femoral fractures. Skeletal Radiol. 2019;48(9):1427-34.
108. Moon NH, Shin WC, Do MU, Woo SH, Son SM, Suh KT. Diagnostic strategy for elderly patients with isolated greater trochanter fractures on plain radiographs. BMC Musculoskelet Disord. 2018;19(1):256.

109. Gatt T, Cutajar D, Borg L, Giordmaina R. The Necessity of CT Hip Scans in the Investigation of Occult Hip Fractures and Their Effect on Patient Management. Advances in Orthopedics. 2021;2021:8118147.
110. Sharrock M, Mati W, Peng Koh S, Abdullah M, Charalambous CP. Additional Imaging is of Limited Value in Traumatic Hip Fractures With a History of Distant Malignancy and No Suspicious Lesion on Plain Radiographs. Journal of Orthopaedic Trauma. 2022;36(11):593-8.
111. Kim J, Yoon HJ, Yoo JJ, Kim HJ. Multiplanar reformation computed tomogram is better than MR imaging in decision making for apparently isolated fractures of the greater trochanter. J Orthop Trauma. 2013;27(8):e181-5.
112. Lee KH, Kim HM, Kim YS, Jeong C, Moon CW, Lee SU, et al. Isolated fractures of the greater trochanter with occult intertrochanteric extension. Arch Orthop Trauma Surg. 2010;130(10):1275-80.
113. Guanche CA, Kozin SH, Levy AS, Brody LA. The use of MRI in the diagnosis of occult hip fractures in the elderly: A preliminary review Orthopedics. 1994;17(4):327-30.
114. Collin D, Geijer M, Göthlin JH. Computed tomography compared to magnetic resonance imaging in occult or suspect hip fractures. A retrospective study in 44 patients. Eur Radiol. 2016;26(11):3932-8.
115. Collin D, Göthlin JH, Nilsson M, Hellström M, Geijer M. Added value of interpreter experience in occult and suspect hip fractures: a retrospective analysis of 254 patients. Emerg Radiol. 2016;23(3):229-34.
